# Whole transcriptome–based skin virome profiling in typical epidermodysplasia verruciformis reveals **α**-, **β**-, and **γ**-HPV infections

**DOI:** 10.1172/jci.insight.162558

**Published:** 2023-03-08

**Authors:** Amir Hossein Saeidian, Leila Youssefian, Mahtab Naji, Hamidreza Mahmoudi, Samantha M. Barnada, Charles Huang, Karim Naghipoor, Amir Hozhabrpour, Jason S. Park, Flavia Manzo Margiotta, Fatemeh Vahidnezhad, Zahra Saffarian, Kambiz Kamyab-Hesari, Mohammad Tolouei, Niloofar Faraji, Seyyede Zeinab Azimi, Ghazal Namdari, Parvin Mansouri, Jean-Laurent Casanova, Vivien Béziat, Emmanuelle Jouanguy, Jouni Uitto, Hassan Vahidnezhad

**Affiliations:** 1 Department of Dermatology and Cutaneous Biology, Sidney Kimmel Medical College, and; 2 Jefferson Institute of Molecular Medicine, Thomas Jefferson University, Philadelphia, Pennsylvania, USA.; 3 Center for Applied Genomics, Children’s Hospital of Philadelphia, Philadelphia, Pennsylvania, USA.; 4 Department of Pathology and Laboratory Medicine, UCLA Clinical Genomics Center, David Geffen School of Medicine at UCLA, Los Angeles, California, USA.; 5 University of California, Riverside, School of Medicine, California, USA.; 6 Department of Dermatology, Razi Hospital, Tehran University of Medical Sciences, Tehran, Iran.; 7 Genetics, Genomics and Cancer Biology PhD Program, Thomas Jefferson University, Philadelphia, Pennsylvania, USA.; 8 Student Research Committee, Faculty of Medicine, Mashhad University of Medical Sciences, Mashhad, Iran.; 9 Department of Medical Genetics and Molecular Biology, Faculty of Medicine, Iran University of Medical Sciences, Tehran, Iran.; 10 Geisinger Commonwealth School of Medicine, Scranton, Pennsylvania, USA.; 11 Department of Dermatology, University of Pisa, Pisa, Italy.; 12 UCSC Silicon Valley Extension, University of California, Santa Cruz, California, USA.; 13 Imam Khomeini Hospital, Tehran University of Medical Science, Tehran, Iran.; 14 Department of Plastic Surgery, School of Medicine, and; 15 Razi Clinical Research Development Unit, Razi Hospital, Guilan University of Medical Science, Rasht, Iran.; 16 Center for Research and Training in Skin Diseases and Leprosy, Tehran University of Medical Sciences, Tehran, Iran.; 17 Department of Dermatology, Ahvaz Jundishapur University of Medical Sciences, Ahvaz, Iran.; 18 Department of Research, Skin and Stem Cell Research Center, Tehran University of Medical Sciences, Tehran, Iran.; 19 St. Giles Laboratory of Human Genetics of Infectious Diseases, Rockefeller Branch, The Rockefeller University, New York, New York, USA.; 20 Laboratory of Human Genetics of Infectious Diseases, Necker Branch, INSERM U1163, Necker Hospital for Sick Children, Paris, France.; 21 Imagine Institute, Paris University, Paris, France.; 22 Department of Pediatrics, Necker Hospital for Sick Children, Paris, France.; 23 Howard Hughes Medical Institute, New York, New York, USA.

**Keywords:** Dermatology, Genetics, Genetic diseases, Molecular diagnosis, Skin

## Abstract

HPVs are DNA viruses include approximately 450 types that are classified into 5 genera (α-, β-, γ-, μ-, and ν-HPV). The γ- and β-HPVs are present in low copy numbers in healthy individuals; however, in patients with an inborn error of immunity, certain species of β-HPVs can cause epidermodysplasia verruciformis (EV), manifesting as recalcitrant cutaneous warts and skin cancer. EV presents as either typical or atypical. Manifestations of typical EV are limited to the skin and are caused by abnormal keratinocyte-intrinsic immunity to β-HPVs due to pathogenic sequence variants in *TMC6*, *TMC8*, or *CIB1*. We applied a transcriptome-based computational pipeline, VirPy, to RNA extracted from normal-appearing skin and wart samples of patients with typical EV to explore the viral and human genetic determinants. In 26 patients, 9 distinct biallelic mutations were detected in *TMC6*, *TMC8*, and *CIB1*, 7 of which are previously unreported to our knowledge. Additionally, 20 different HPV species, including 3 α-HPVs, 16 β-HPVs, and 1 γ-HPV, were detected, 8 of which are reported here for the first time to our knowledge in patients with EV (β-HPV-37, -47, -80, -151, and -159; α-HPV-2 and -57; and γ-HPV-128). This study expands the *TMC6*, *TMC8*, and *CIB1* sequence variant spectrum and implicates new HPV subtypes in the pathogenesis of typical EV.

## Graphical abstract

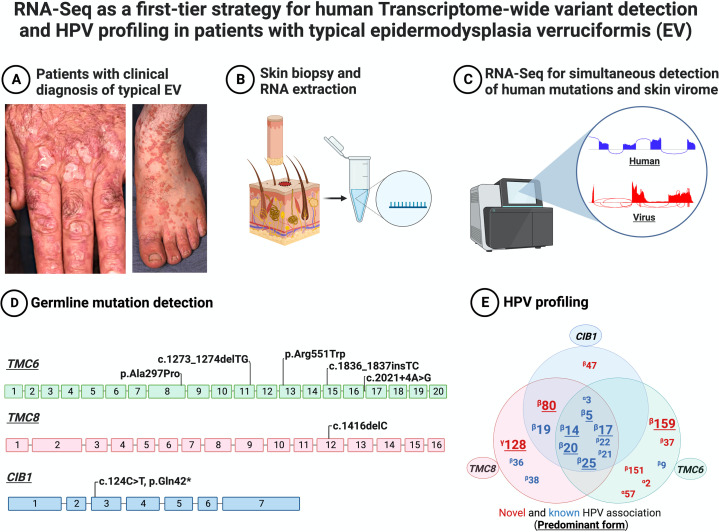

## Introduction

HPVs are a ubiquitous and diverse group of DNA viruses and include approximately 450 HPV types classified into 5 genera (α-, β-, γ-, μ-, and ν-HPV). Whereas α-HPVs display cutaneous and mucosal tropism, other genera strictly infect cutaneous tissue. With the advent of sensitive PCR methodologies and DNA-based next-generation sequencing (NGS) technologies, it was discovered that at least 50 different cutaneous HPVs, belonging to the γ-HPV and β-HPV genera, are present in low copy numbers in healthy individuals from infancy, leading to subclinical infections. By using DNA-based NGS, these γ- and β-HPVs can be detected in the skin of 45% of healthy infants and in 80% of healthy adults; as such, they can be considered commensal organisms. The copy number and diversity of γ- and β-HPVs are greater among organ transplant recipients, patients with a secondary immunodeficiency, and patients with an inborn error of immunity (IEI), and occasionally lead to the appearance of recalcitrant warts (1, 2).

The prototypical recalcitrant wart is epidermodysplasia verruciformis (EV), a subset of cutaneous warts caused by chronic infection with specific types of β-HPVs (3, 4). Infection with γ-HPVs in EV was suspected in 1 early case (5). EV is an exceedingly rare entity and presents as either typical or atypical; typical EV is invariably limited to cutaneous HPV infection without extracutaneous manifestations. To date, biallelic loss-of-function (LoF) sequence variants in 3 genes, *TMC6*, *TMC8*, and *CIB1*, have been associated with the pathogenesis of typical EV. In contrast, in patients with IEI, atypical EV presents with additional extracutaneous infections due to LoF mutations in more than 10 genes expressed in T lymphocytes (6). The clinical manifestations of EV consist of pityriasis versicolor–like lesions and widespread, persistent, flat warts (7). These lesions typically appear during early childhood, particularly on sun-exposed areas, and often evolve into nonmelanoma skin cancers (NMSCs) in 30%–70% of patients with EV due to persistent HPV infections (8). Although the prevailing literature has suggested that β-HPV-5 and -8 are the predominant EV-causing HPVs found in these patients, no comprehensive study appears to have confirmed this hypothesis (9, 10).

**Figure 1:**
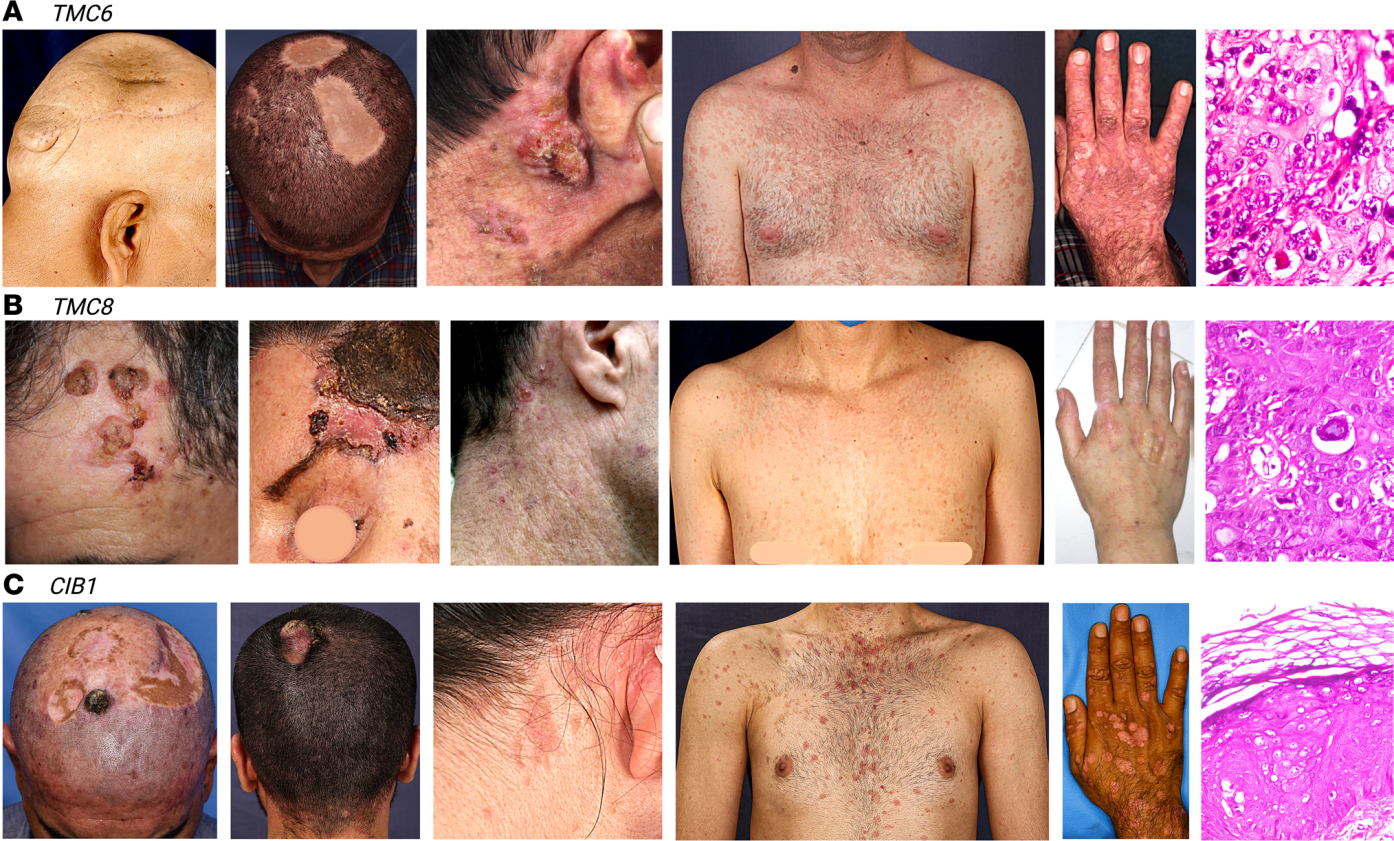
Phenotypic spectrum in typical EV. (**A–C**) Note the presence and similarity of flat verrucous lesions, verrucous tumors, and SCCs in all patients with mutations in *TMC6*, *TMC8*, and *CIB1*. Histopathology obtained from the biopsied skin of patients with typical EV commonly showed large cells with blue-gray cytoplasm with keratohyalin granules in both the granular and spinous layers, reflecting HPV infection in the skin of the patients. Created with BioRender.com.

**Figure 2:**
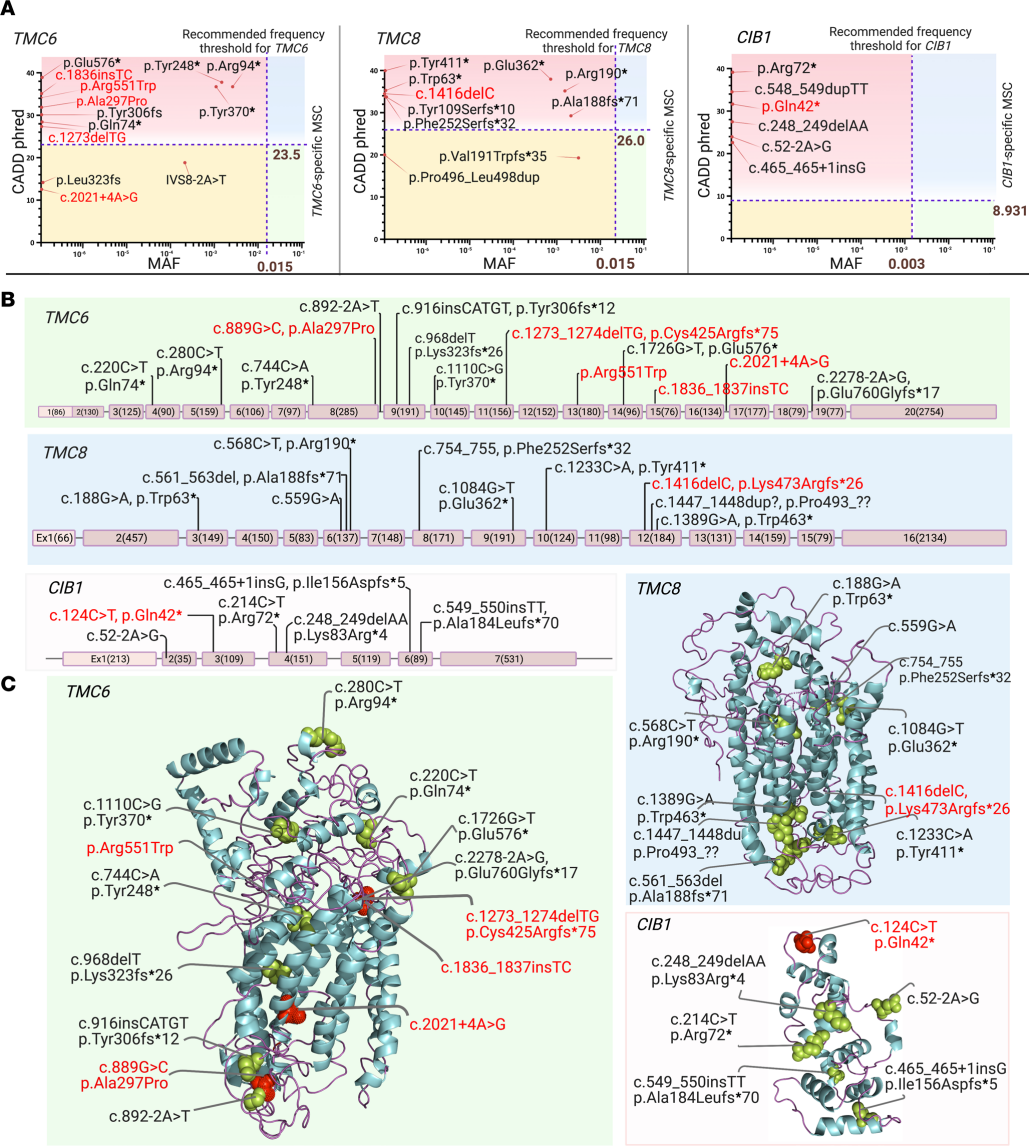
Gene and protein structures of *TMC6*, *TMC8*, and *CIB1*, and the positions of pathogenic mutations. (**A**) Plots of CADD score versus minor allele frequency (MAF) of all previously reported (black) and potentially novel (red) variants in *TMC6*, *TMC8*, and *CIB1*. Although the CADD score of 20 for any given sequence variant indicates the theoretical top 1% of deleterious variants, the *TMC6*, *TMC8*, and *CIB1* gene-specific MSC with 99% CIs was 23.5, 26, and 8.931, respectively. The recommended gene-specific MAF thresholds for *TMC6*, *TMC8*, and *CIB1* are 0.015, 0.015, and 0.003, respectively. There are 4 groups for variants on each plot: (a) rare variants with CADD score greater than the MSC of each gene and MAF less than the gene-specific MAF (red region, top left); (b) rare variants with CADD score less than the MSC of each gene and MAF less than the gene-specific MAF (light yellow region, bottom left); (c) variants with CADD score greater than the MSC of each gene and MAF greater than the gene-specific MAF (blue region, top right); (d) common variants with CADD score less than the MSC of each gene and MAF greater than the gene-specific MAF (green region, bottom right). (**B**) Gene structure and the exon numbers with their sizes (bps) are indicated in parentheses. The introns are not drawn to scale. (**C**) 3D protein structures and the location of variants are shown, generated with PyMOL software. The mutations we believe to be previously unreported are indicated in red. Created with BioRender.com.

In this study, we implemented VirPy, an unbiased transcriptomic approach (11), to data from a cohort of 26 patients with typical EV harboring *TMC6*, *TMC8*, or *CIB1* mutations (Figures 1 and 2). This innovative RNA-based NGS method successfully detected the presence of many of the approximately 450 HPV subtypes, differentiated between active and latent states of HPV infection, and captured the human genetic determinant of typical EV in these patients.

## Results

### Whole-transcriptome sequencing as a first-tier method for concomitant mutation and viral detection.

In this study, we used whole-transcriptome sequencing (RNA-Seq) as the initial sequencing method to study a cohort of 50 patients with typical and atypical EV. In the cases in which RNA-Seq results were unyielding, DNA samples extracted from blood were submitted for whole-exome sequencing (WES). RNA-Seq data aligned with homozygosity mapping (HM) allowed us to identify underlying disease-causing variants in *TMC6*, *TMC8*, and *CIB1* associated with typical EV in 26 patients from 13 distinct consanguineous families. We found 9 distinct variants in *TMC6*, *TMC8*, or *CIB1*, 7 of which were previously unreported to our knowledge: 5 in *TMC6*, 1 in *TMC8*, and 1 in *CIB1* (Figure 2, A–C). The positions of the mutations found in the 26 patients with typical EV were mapped onto their 3D protein structures using PyMOL (Figure 2C). The remaining 24 patients had mutations in genes related to atypical EV, other genetic etiologies, or remained undiagnosed. Because of the extensive consanguinity in our cohort, all candidate genes were selected on the basis of their presence within regions of homozygosity (ROH). Additionally, Sanger sequencing of genomic DNA from patients and obligate carriers in the families was performed to confirm the segregation of the mutated gene. We used established bioinformatics algorithms for pathogenicity prediction (12) (e.g., sorting intolerant from tolerant [SIFT]; Polymorphism Phenotyping, version 2 [PolyPhen2]; combined annotation-dependent depletion [CADD]; MetaLR [https://useast.ensembl.org/info/genome/variation/prediction/protein_function.html]; Mendelian Clinically Applicable Pathogenicity [M-CAP]; Primate Al; rare exome variant ensemble learner [REVEL]; VARITY [http://varity.varianteffect.org/]). Allele frequencies were verified using publicly available databases for healthy control participants (13) (e.g., Genome Aggregation Database [gnomAD] Aggregated, Trans-Omics for Precision Medicine [TOPMed], BRAVO [https://bravo.sph.umich.edu/freeze8/hg38/], Iranome, Greater Middle East [GME] Variome).

### Five TMC6 variants in EV families with extensive NMSC.

Patients with typical EV frequently develop NMSCs, predominantly on the sun-exposed areas of skin, with squamous cell carcinoma (SCC) being more frequent than basal cell carcinoma (BCC) (6). NMSC was found in 22 of 26 patients with typical EV (84%); specifically, 14 had SCC, 5 had BCC, and 3 had both SCC and BCC, all with differing HPV repertoires (mean [SD] age, 27.6 [10.7] years; 40% women) (Tables 1–3).

**Figure 3:**
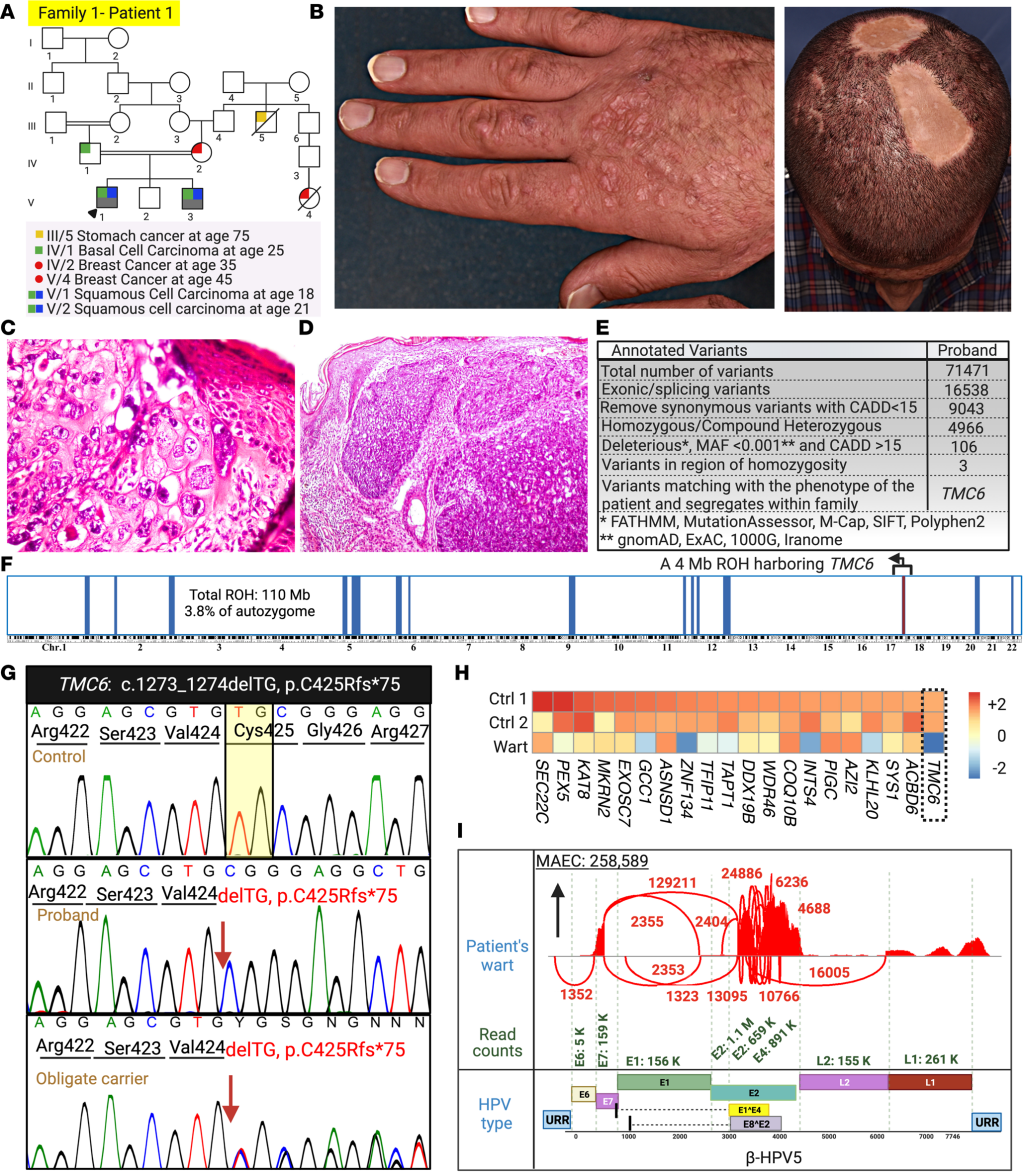
Family 1 pedigree, clinical manifestations, histopathology, genetic etiology, and virome study results. (**A**) Patient 1 is from a multiplex consanguineous family affected by several malignancies, including BSC and SCCs. (**B**) Extensive flat warts on the dorsum of the right hand (same patient as shown in Figure 1A). Note the scars that formed after surgical excision of BCC and SCC. (**C**) Presence of koilocytes within a wart lesion indicative of HPV infection. (**D**) Histopathology of the patient’s SCC sample. (**E** and **F**) RNA-Seq data and HM revealed a possibly novel homozygous variant in *TMC6*: c.1273_1274delTG, p.Cys425Argfs*75 in a 4 Mb ROH harboring the *TMC6* gene. (**G**) The mutation was confirmed by Sanger sequencing. (**H**) The gene expression level for *TMC6* mRNA was significantly reduced compared with healthy control participants. (**I**) We detected β-HPV-5 in the proband’s wart biopsy by VirPy. Created with BioRender.com. 1000G, 1000 Genomes Project; ExAC, Exome Aggregation Consortium; URR, upstream regulatory region.

Patient 1 exhibited extensive, flat, wart-like lesions with BCCs and SCCs at an early age. The patient’s father developed a BCC at age 25 years, and his mother developed breast cancer at age 35 years (Figure 3, A–D). We found a homozygous sequence variant, *TMC6*: c.1273_1274delTG, p.Cys425Argfs*75 (Figure 2 and Figure 3, E–G), which was absent in all publicly available control databases. The *TMC6* mRNA expression level was significantly reduced in patient 1 compared with that of healthy control participants, whereas housekeeping genes were not dysregulated (Figure 3H). VirPy detected β-HPV-5 in the proband’s wart biopsy specimen (Figure 3I). Additionally, given the high prevalence of cancer within this family, known cancer-associated genes were referenced, and a heterozygous pathogenic variant, *MLH1*: c.204_205del, p.Thr69Lysfs*2, was found.

**Figure 4:**
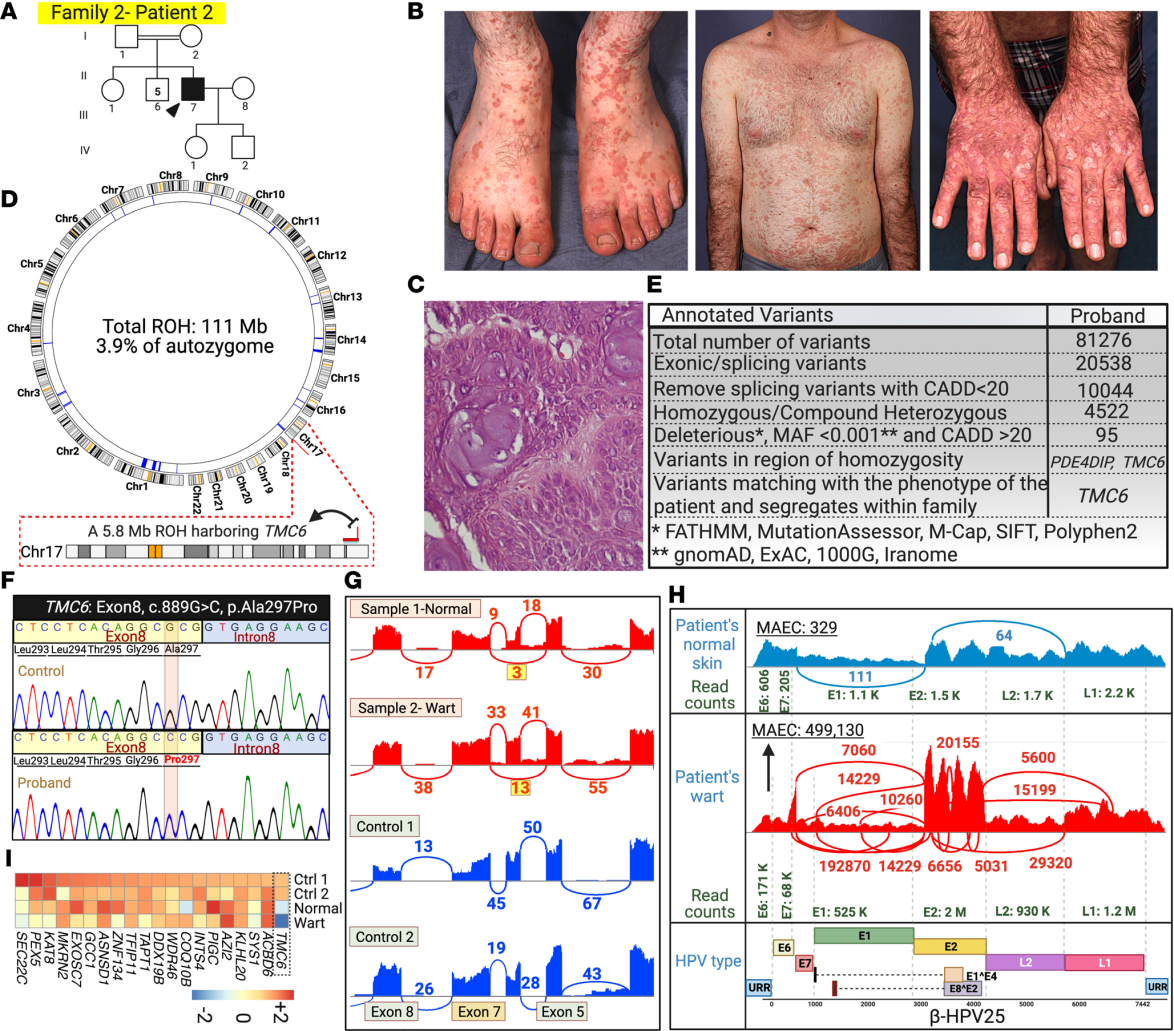
Family 2 pedigree, clinical manifestations, histopathology, genetic etiology and skin virome study results. (**A**) The patient was born to consanguineous parents and has had warts since he was 5 years old. He was also diagnosed with papillary thyroid carcinoma at age 42 years. (**B**) Extensive flat warts throughout his body (same patient as shown in Figure 1A). (**C**) Pathology confirmed the presence of koilocytes in the biopsied wart. (**D**) There was a total of 111 Mb ROH greater than 4 Mb in the patient, and the *TMC6* gene was located within a 5.8 Mb ROH. (**E** and **F**) RNA-Seq analysis and Sanger sequencing revealed a homozygous missense variant with potentially damaging effects on splicing in *TMC6*: c.889G>C, p.Ala297Pro. (**G**) Sashimi plot shows partial skipping of exon 6 downstream of the variant in both normal-appearing skin and a wart of the patient. (**H**) VirPy detected high levels of β-HPV-25 in the wart sample and lower levels in the patient’s normal-looking skin. (**I**) *TMC6* mRNA expression level was reduced compared with that of healthy control participants. Created with BioRender.com. 1000G, 1000 Genomes Project; ExAC, Exome Aggregation Consortium; URR, upstream regulatory region.

The proband of family 2, with 2 healthy children, has had warts since age 5 years, and contents of fine-needle aspiration of a thyroid nodule at age 42 years showed papillary thyroid carcinoma (Figure 4, A and B). Histopathology confirmed the presence of koilocytes in a biopsied wart sample (Figure 4C). A homozygous missense variant with potentially damaging splicing effects, *TMC6*: c.889G>C, p.Ala297Pro (rs750652637), was found in the sequence encoding the final amino acid of exon 8 (Figure 2 and Figure 4, D–G). To our knowledge, this is the first time this missense/splicing mutation has been reported in *TMC6*. This variant has not been reported as homozygous in healthy individuals in publicly available databases.

On the basis of various available algorithms for missense pathogenicity prediction (including SIFT, Polyphen 2, VARITY, and Functional Analysis through Hidden Markov Models [FATHMM]), the rs750652637 sequence variant was determined to be pathogenic, with a CADD score of 32, well above the mutation significance cutoff (MSC) of 22.4 for *TMC6* (Figure 2). The *TMC6* mRNA level was reduced in both normal-appearing skin and wart samples of patient 2, compared with that of skin samples from healthy control participants. (Figure 4I). Interestingly, despite the Human Splicing Finder (HSF) (http://umd.be/Redirect.html) prediction of “no potential impact on splicing” for this missense variant, the Sashimi plot showed partial skipping of exon 6 in both the wart and normal-appearing skin samples of the patient (Figure 4G). VirPy detected β-HPV-25 in high amounts and β-HPV-14 in very low amounts in both wart and normal-appearing skin, and β-HPV-37 was detected in low amounts in the normal-appearing skin only (Figure 4H and Table 1).

**Figure 5:**
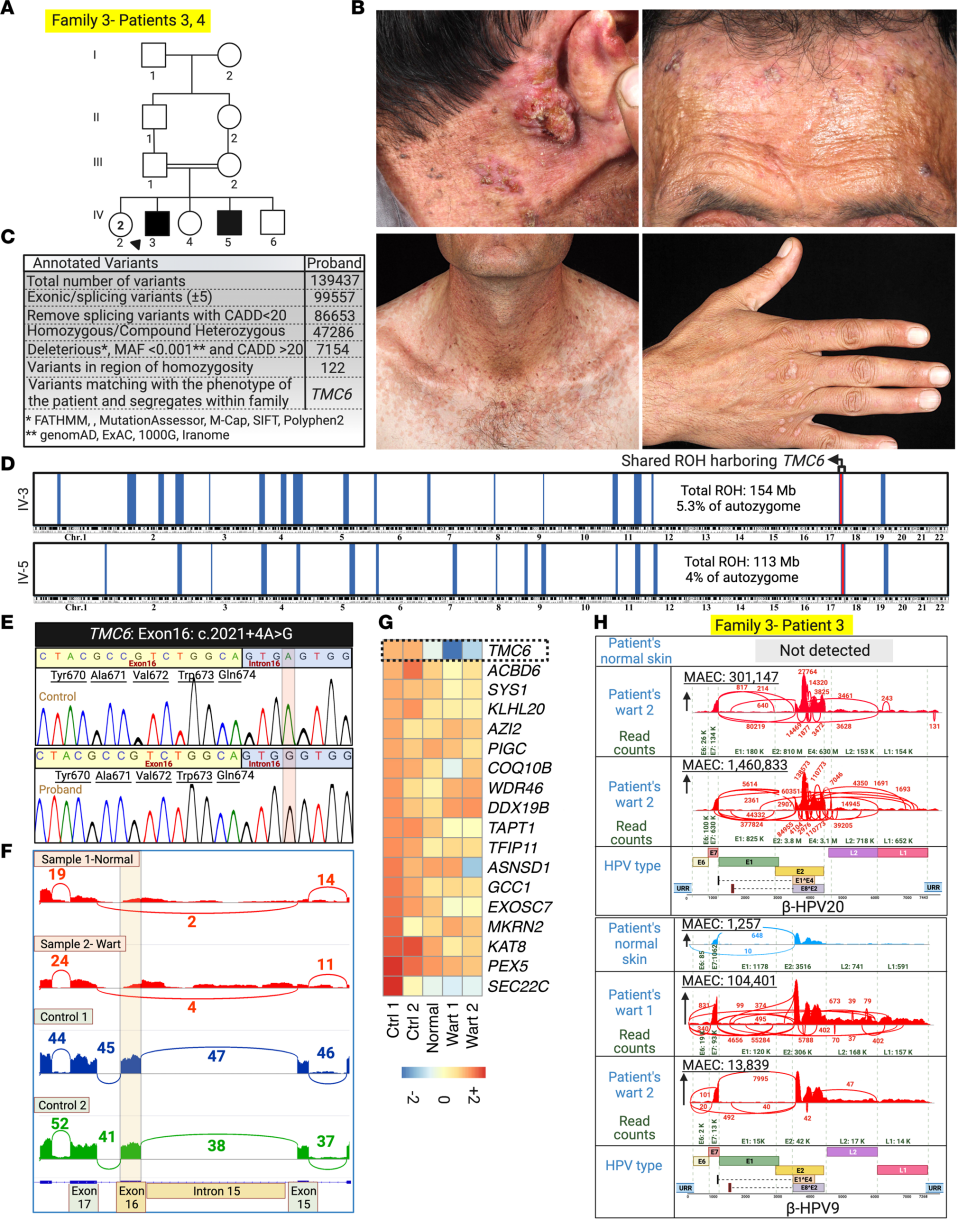
Family 3 pedigree, clinical manifestations, and whole-transcriptome analysis for human genetic and skin virome study. (**A** and **B**) The proband and his affected brother, born to consanguineous parents, showed multiple extensive flat warts (same patient as shown in Figure 1A). The proband has had multiple SCCs since age 30 years on different parts of his body, including the posterior auricular area, forehead, and neck. (**C** and **D**) RNA-Seq data and HM revealed a noncanonical splice site variant in this family, *TMC6*: c.2021+4A>G, located within a homozygous block shared by both patients. (**E**) Sanger sequencing confirmed the noncanonical splice site mutation. (**F**) Sashimi plot shows the skipping of exon 16 in the RNA-Seq results of both normal-appearing skin and wart biopsy specimens of the proband. (**G**) *TMC6* mRNA level was reduced compared with that of healthy control participants. (**H**) We detected β-HPV-20 and -9 in the warts of both patients. Comparatively lower levels of β-HPV-9 were detected in the normal-appearing skin. Created with BioRender.com. 1000G, 1000 Genomes Project; ExAC, Exome Aggregation Consortium; URR, upstream regulatory region.

The proband and his affected brother in family 3 presented with multiple, extensive flat warts. Multiple SCCs on the posterior auricular area, forehead, and neck were observed in the proband since 30 years of age (Figure 5, A and B). We found a noncanonical variant of uncertain significance (VUS), *TMC6*: c.2021+4A>G, that resided within an ROH common to both patients on chromosome 17, potentially affecting splicing (Figure 2 and Figure 5, C–E). The Sashimi plot and Sanger sequencing of cDNA showed skipping of exon 16 in patients 3 and 4 (Figure 5F). The *TMC6* mRNA level was reduced in both normal-appearing skin and wart samples of patient 3 compared with that of skin samples from healthy control participants (Figure 5G). Using VirPy, we detected β-HPV-20 and β-HPV-9 in both patients’ warts. A low level of β-HPV9 was also detected in their normal-appearing skin (Figure 5H and Table 1).

**Figure 6:**
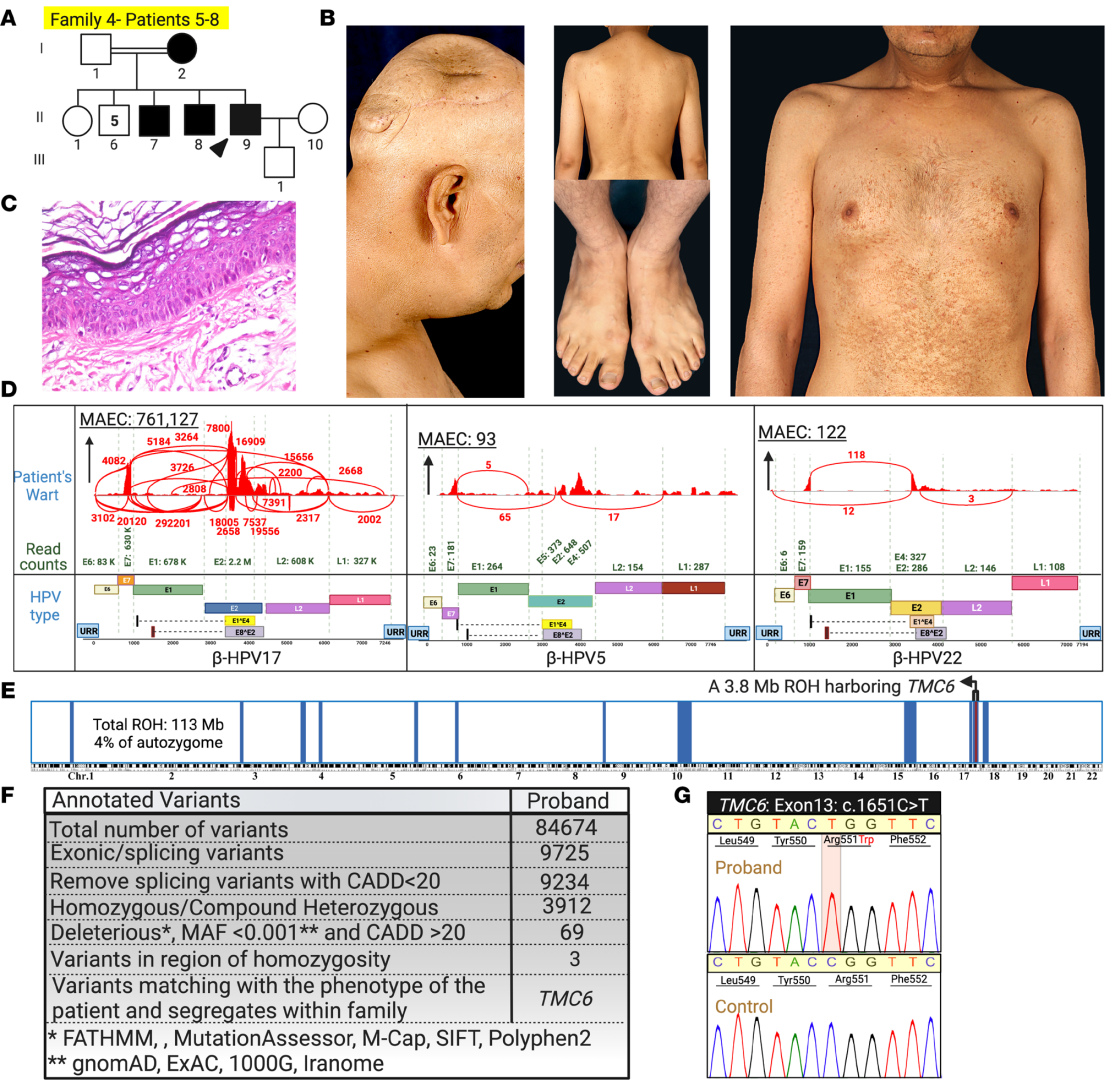
Family 4 pedigree, clinical manifestations, histopathology, and whole-transcriptome analysis for human genetic and skin virome study. (**A** and **B**) The proband, a 40-year-old man with a consanguineous background, has extensive flat warts. Please note the SCC lesions of the same patient, shown in Figure 1, which were removed from his scalp when he was 36 years old. (**C**) The presence of koilocytes and hyperkeratosis was consistent with EV diagnosis. (**D**) β-HPV-17 was detected at much higher levels than β-HPV-5 and -22 in this patient. (**E** and **F**) RNA-Seq data and HM revealed a homozygous *TMC6*:c1651C>T, p.Arg551Trp VUS. (**G**) Sanger sequencing confirmed this missense mutation. Created with BioRender.com. 1000G, 1000 Genomes Project; ExAC, Exome Aggregation Consortium; URR, upstream regulatory region.

The proband of family 4 was a 40-year-old man with flat warts, and examination of a skin biopsy specimen confirmed the presence of koilocytes. Multiple SCCs were surgically removed from his scalp when he was 36 years old (Figure 6, A–C). We documented a homozygous missense variant, *TMC6*: c.1651C>T, p.Arg551Trp, with a CADD score of 33 and a VARITY_ER_LOO score of 0.943 (Figure 2 and Figure 6, E–G) (14). The arginine residue at this position is evolutionarily conserved. This VUS was present in population databases (rs779481795; gnomAD 0.001%) as heterozygous (*n* = 3) but does not appear to have been reported in the homozygous state. The HSF predicted a “significant alteration of exonic splicing enhancer/silencer motifs ratio” of –4 for this missense variant. β-HPV-17 was the only HPV detected at clinically relevant levels in this patient (Figure 6D and Table 1).

**Figure 7:**
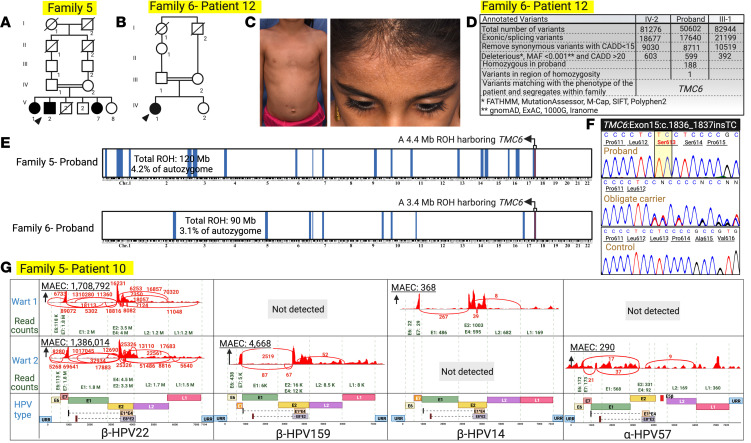
Pedigrees of families 5 and 6, clinical manifestations, genetic analysis, and skin virome results. (**A–C**) The probands of family 5 and family 6 were from consanguineous backgrounds and had widely distributed flat warts. (**D–F**) We found a homozygous 2 bp insertion mutation in *TMC6*: c.1836_1837insTC in both families. This mutation resided within a shared homozygosity block. Sanger sequencing confirmed this homozygous frameshift mutation. (**G**) We detected β-HPV-14, -22, and -159, and α-HPV-57 in the skin biopsy specimens of the proband of family 5. The proband in family 6 did not consent to virome evaluation. Created with BioRender.com. 1000G, 1000 Genomes Project; ExAC, Exome Aggregation Consortium; URR, upstream regulatory region.

Mutation detection by RNA-Seq for the proband of family 5 and WES for the proband of family 6 revealed a homozygous insertion mutation, *TMC6*: c.1836_1837insTC, located within an ROH shared by both patients (Figure 2 and Figure 7, A–G). VirPy detected 4 different HPVs — β-HPV-14, -22, and -159, and α-HPV-57 — in the skin biopsy specimens of the proband of family 5 (Figure 7G and Table 1). The proband in family 6 did not consent to virome evaluation.

### Association of γ-HPV-128 and β-HPV-80 with typical EV in a family with a TMC8 variant.

**Figure 8:**
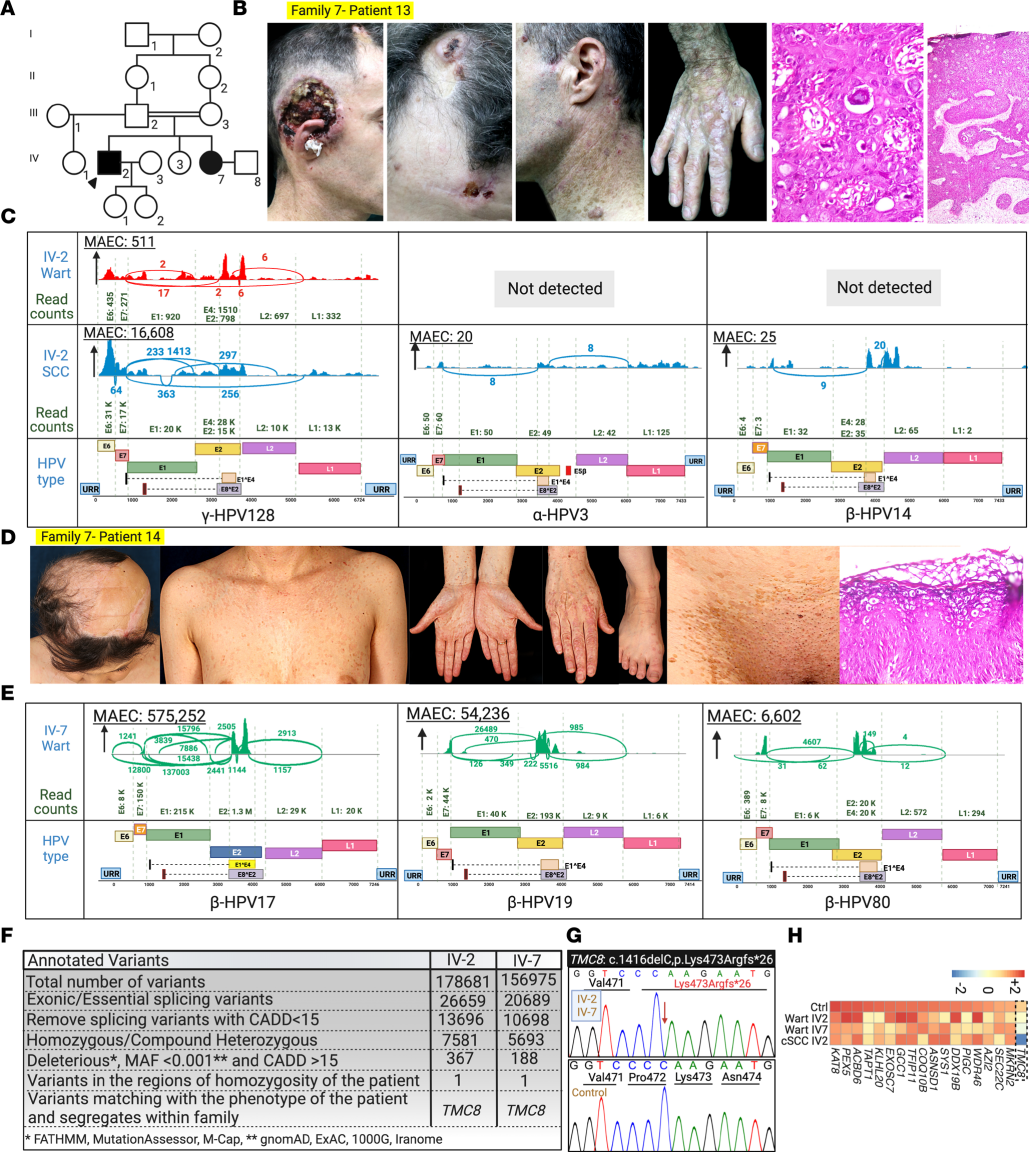
Family 7 pedigree, clinical manifestations, virome, and genetics, with the first association of γ-HPV-128 in EV patient with *TMC8* variant. (**A**) Pedigree of family 7. Note the consanguinity between the parents of the 2 affected individuals. (**B** and **D**) The same patient is shown in Figure 1B. The clinical presentation of the proband (IV-2) (**B**) and his sister (IV-7) (**D**). Note the presence of malignant SCC, multiple pityriasis versicolor–like warts, and koilocytes in the histopathology. (**C**) Sashimi plots derived from RNA-Seq data, delineating the types of HPVs associated with the wart and SCC of the proband. Note the presence of γ-HPV-128 in both the wart and SCC. Lower levels of α-HPV-3 and β-HPV-14 were also detected in the SCC sample. (**E**) Sashimi plots identifying β-HPV-17, -19, and -80 in the proband’s sister (IV-7). (**F**) Stepwise bioinformatic filtering was performed, and the mutation in *TMC8*: c.1416delC was detected. (**G**) Sanger sequencing confirmed the presence of the *TMC8*: c.1416delC in homozygous state. (**H**) Heatmap of the differential expression of *TMC8* and other housekeeping genes in warts and SCC lesions in family 7 compared with those of a healthy control participant. *TMC8* expression levels were noticeably reduced in both wart and SCC lesions in family 7 compared with a healthy control participant. Created with BioRender.com. 1000G, 1000 Genomes Project; Exome Aggregation Consortium; URR, upstream regulatory region.

Two patients in family 7, a 41-year-old man and his 27-year-old sister, had extensive recalcitrant warts and SCCs (Figure 8, A, B, and D). In both patients, we detected a homozygous variant, which appears to be previously unreported, within a shared 6 Mb ROH: *TMC8*: c.1416delC, p.Lys473Argfs*26 (Figure 2 and Figure 8, F and G), with a CADD score of 35, well above the MSC of 26 (Figure 2). This variant led to the depletion of *TMC8* mRNA (Figure 8H). γ-HPV-128 was the predominant HPV in the proband’s wart and SCC lesions (Figure 8C). To our knowledge, this is the first report of the association of γ-HPV-128 with typical EV. Interestingly, γ-HPV-128 was not detected in the proband’s sister, who lived in a different household; however, β-HPV-17, β-HPV-19, and a possibly previously unreported β-HPV-80 were detected (Figure 8E and Table 2). Because β-HPV-80 and γ-HPV-128 appear not to have been associated previously with patients with typical EV, we validated these findings and the presence of various other common HPV types by a conventional RT-PCR method (Supplemental Figure 1; supplemental material available online with this article; https://doi.org/10.1172/jci.insight.162558DS1).

**Figure 9:**
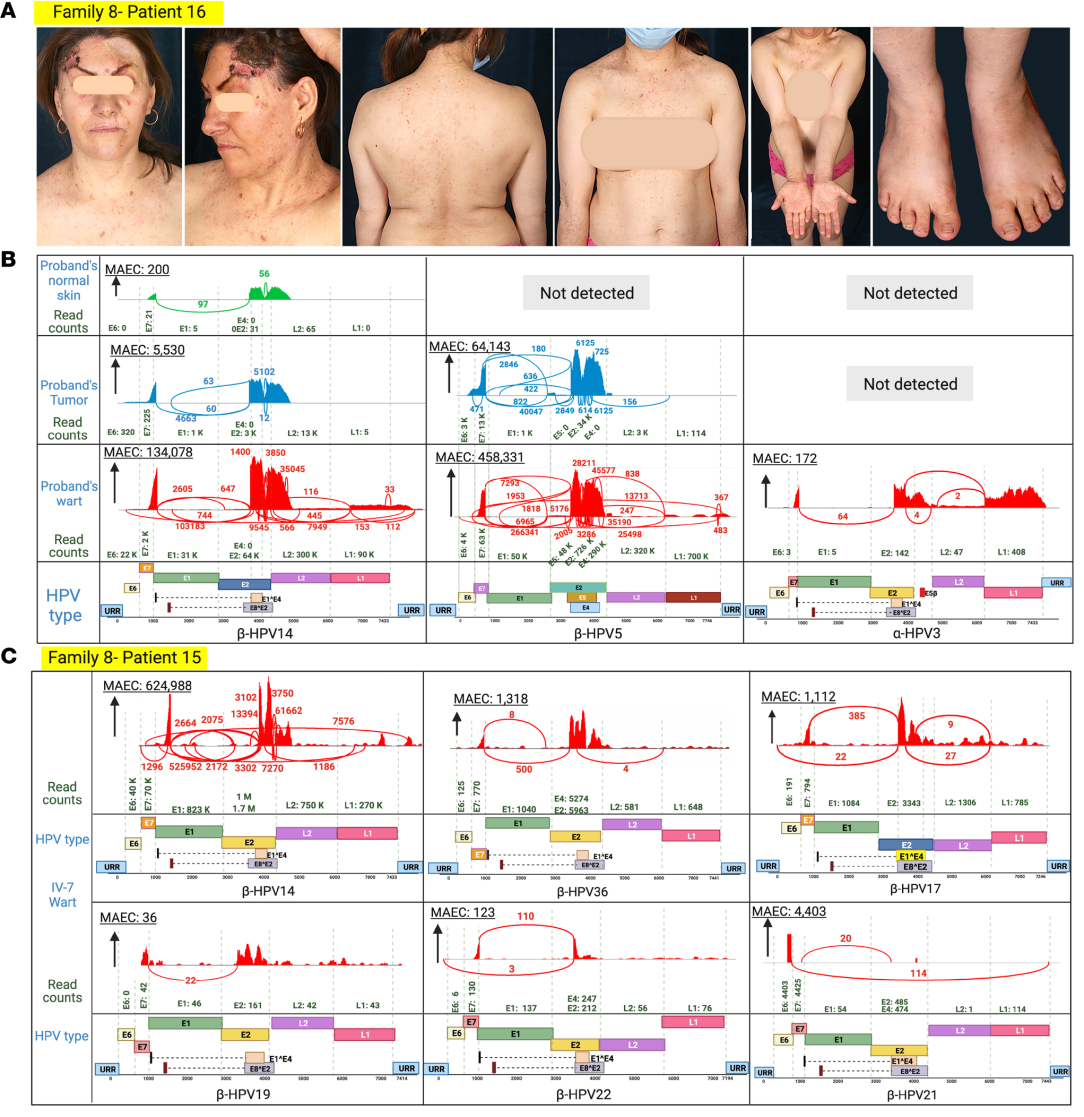
Family 8 clinical manifestations, and skin virome study. (**A**) Same patient as shown in Figure 1B. Family members had flat warts all over their body, and the patient also had an SCC excised from the scalp. (**B**) Using VirPy, we found β-HPV-14 and -5 were the predominant HPV subtypes. Low levels of α-HPV-3 were also detected in the proband. (**C**) The sibling was positive for multiple HPVs, including β-HPV-14, -19, -17, -21, -22, and -36. Created with BioRender.com. URR, upstream regulatory region.

**Figure 10:**
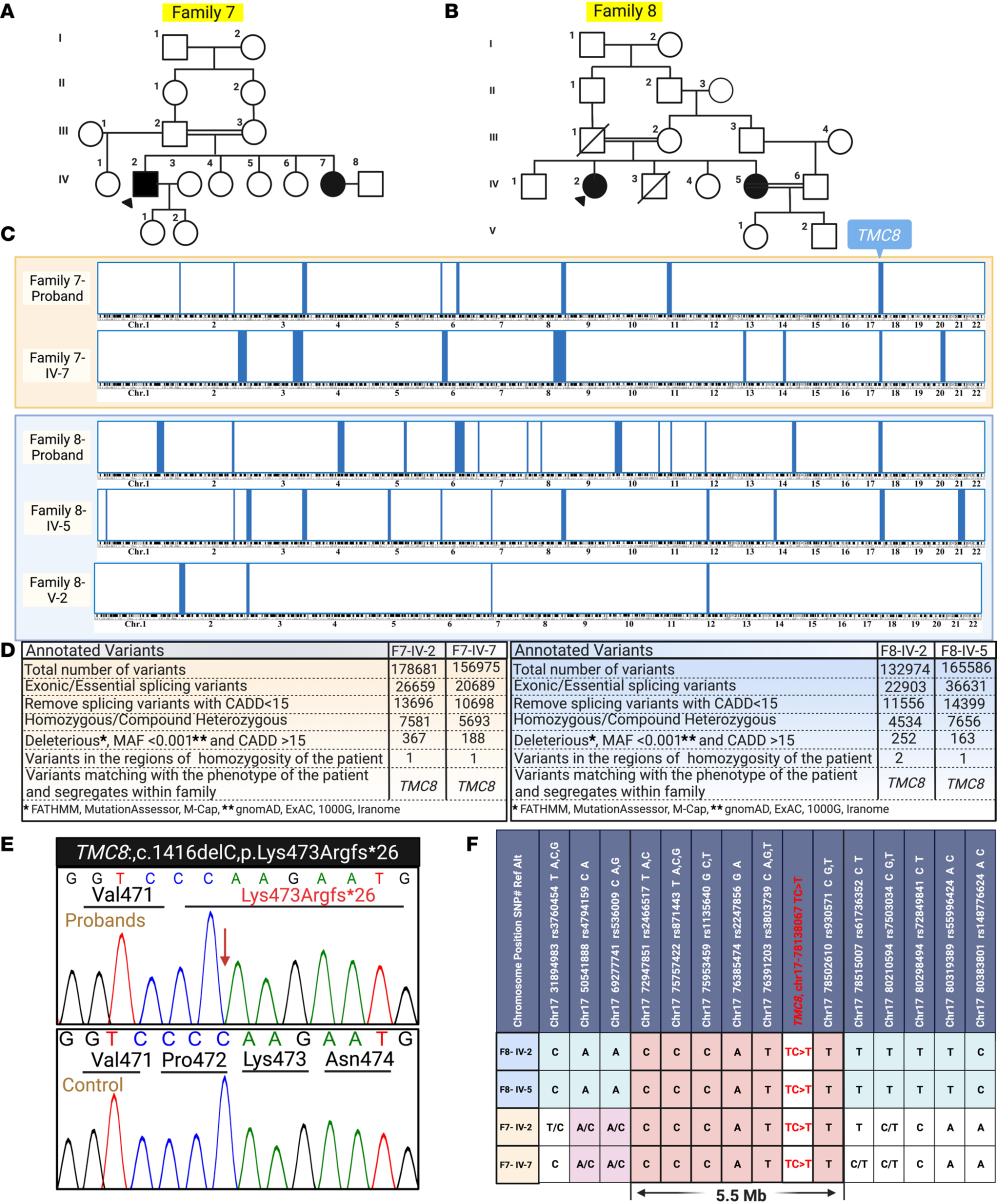
Pedigrees of families 7 and 8, HM, genetic analysis, and haplotype mapping for a *TMC8* founder-effect mutation. (**A** and **B**) The probands of families 7 and 8 were from consanguineous backgrounds. (**C** and **D**) RNA-Seq analysis and HM revealed what we believe to be a previously unreported homozygous variant in *TMC8*: c.1416delC, p.Lys473Argfs*26, located within an ROH shared by all affected patients. This variant was absent in a healthy member of family 8. (**E**) Sanger sequencing confirmed this homozygous deletion mutation. (**F**) Haplotype analysis showed the conserved sequence of SNV tags surrounding the *TMC8* mutation among the 4 affected patients from the 2 families, indicating the founder effect by showing the 5.5 Mb shared haplotype. Created with BioRender.com. 1000G, 1000 Genomes Project; Chr, chromosome; Exome Aggregation Consortium.

The proband of family 8 had an SCC on the fourth digit of her left hand. She and her sister both had an SCC on their scalp (Figure 9A). Family 8 harbored the same *TMC8* variant found in family 7 (Figure 10, A–E). Considering the distant geographic location of these families, we examined the possibility of a founder effect. Haplotype analysis showed the conservation of single nucleotide variants (SNVs) surrounding the *TMC8* mutation among all affected individuals, indicating the founder effect through a 5.5 Mb shared haplotype (Figure 10F). Furthermore, VirPy identified β-HPV-14 and β-HPV-5 as the predominant pathogenic viruses. Low levels of α-HPV-3 were also detected in the proband of family 8 (Figure 9B). However, her younger sibling was positive for multiple HPVs, including β-HPV-14, -17, -19, -21, -22, and -36 (Figure 9C and Table 2).

### Founder effect of CIB1 mutations in 2 families with typical EV.

We previously characterized 7 families with typical EV with LoF mutations in the *CIB1* gene (15); this study adds 5 additional families with 3 distinct pathogenic sequence variants in *CIB1*, including 1 that appears to be previously unreported. Interestingly, the patients in families 9 and 10 who manifested with multiple BCCs and extensive flat warts harbored an identical pathogenic variant in *CIB1*, which also, to our knowledge, is previously unreported.

**Figure 11:**
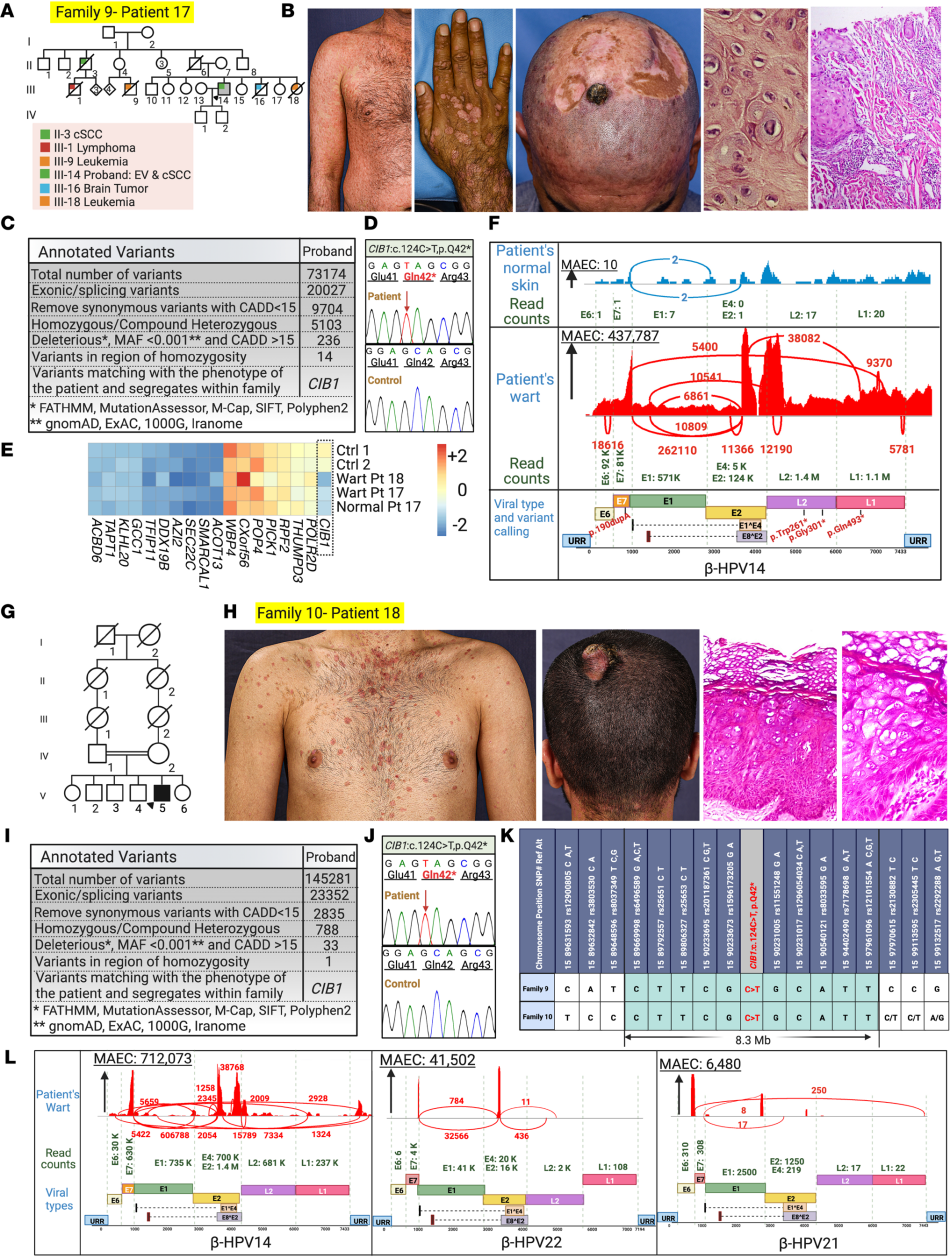
A homozygous nonsense *CIB1* founder mutation shared across 2 unrelated families with typical EV. (**A** and **G**) Family pedigrees and clinical features of patients with *CIB1* mutation. Note the parental consanguinity and multiple hematological and cutaneous malignancies within the extended families. (**B** and **H**) Multiple confluent pityriasis versicolor–like warts and BCC in the probands were observed, as shown in Figure 1C. Histopathology of the lesions showed orthokeratosis, hypergranulosis, and papillomatosis with dyskeratotic cells, and some keratinocytes of the upper squamous layer had vacuoles in their cytoplasm with nuclear inclusion, consistent with the diagnosis of HPV infection. Excisional biopsy from scalp lesion showed atypical squamoproliferative tumor with invasive buds (upper right panel). (**C** and **I**) Stepwise bioinformatic filtering on the data generated from RNA-Seq reduced the number of candidate variants leading to the identification of *CIB1* as the candidate gene. (**D** and **J**) The mutation, *CIB1*: NM_006384.3, Exon 3, c.124C>T, p.Gln42*, was confirmed as homozygous in the probands through Sanger sequencing. (**E**) Heatmap expression profiling of RNA-Seq data displays significantly reduced *CIB1* mRNA levels in the proband of family 9, compared with randomly selected housekeeping genes and other EV-associated genes, suggesting nonsense-mediated mRNA decay. (**F** and **L**) Sashimi plot (patient 17) showing the identification and quantification of full-length β-HPV-14, via VirPy, with expression in the wart and no expression in normal-appearing skin biopsy specimens of the proband. β-HPV-14, -22, and -21 were detected in the wart of patient 18. (**K**) To determine whether the mutation detected in these 2 families is a hot-spot mutation or a founder-effect mutation, we compared rare homozygous SNVs surrounding the *CIB1* locus in chromosome 15, region q26.1, called from RNA-Seq data. Note conservation of the polymorphic markers within an 8.3 Mb block of DNA flanking the *CIB1* locus. Created with BioRender.com. 1000G, 1000 Genomes Project; Exome Aggregation Consortium; URR, upstream regulatory region.

The proband of family 9 was a 50-year-old man with recalcitrant warts on his hands, neck, and back that appeared when he was approximately 10 years old. Multiple members of the extended family had hematologic malignancies (Figure 11, A and B). Histopathology of wart samples was consistent with HPV infection (Figure 11B). The patient underwent 6 surgeries for tumor removal, and a specimen from an excisional biopsy of a scalp lesion showed an atypical squamoproliferative tumor with invasive buds. The proband of family 10 was a 33-year-old man with extensive warts and an SCC on his scalp (Figure 11, G and H). In both families, we found a homozygous nonsense variant, *CIB1*: NM_006384.3, Exon3, c.124C>T, p.Gln42*, with a CADD score of 34, well above the MSC of 3.313 specific for *CIB1* (Figure 2A and Figure 11, C–E, I, and J). This *CIB1* variant was located within a 42 Mb ROH and a 6.7 Mb ROH in the probands of families 9 and 10, respectively.

Furthermore, the alignment of known EV-associated gene loci with HM showed that *CIB1* was the only gene co-aligning with shared ROHs in both patients (Figures 11, C and I). The allelic frequencies of this variant, both homozygous and heterozygous, in 250,242 alleles of healthy individuals (gnomAD r2.1) were 0 and 1, respectively, yielding an allele frequency of 0.0004%. We found this mutation within shared blocks of SNV tags spanning 8.3 Mb between these unrelated patients, which indicated a founder-effect mutation (Figure 11K). Using VirPy, we found β-HPV-14 in the proband of family 9 and β-HPV-14, -21, and -22 in the proband of family 10 were the predominant HPV subtypes. Despite the presence of more than 1 HPV subtype in the wart sample of patient 18, compared with the singular HPV subtype found in patient 17, no significant difference in phenotypes was seen (Figure 11, F and L, and Table 3).

**Figure 12:**
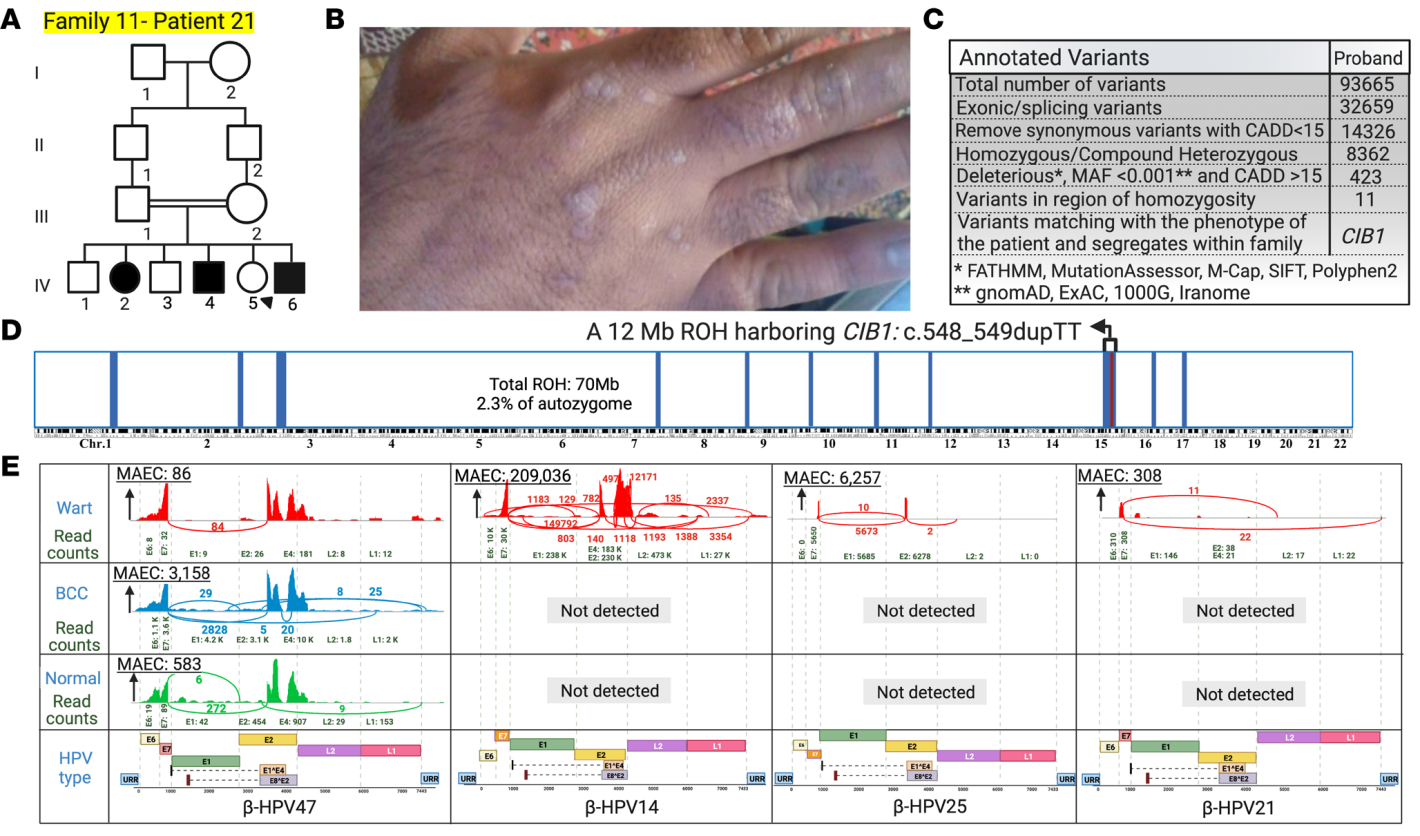
Family 11 pedigree, HM, genetic analysis, and skin virome profiling. (**A**) The proband of family 11 is a 31-year-old man born to first-cousin parents. (**B**) He has extensive flat warts on his body and hands. (**C** and **D**) RNA-Seq analysis and HM revealed a homozygous LoF variant in *CIB1*: NM_006384.3, Exon 6, c.548_549dupTT located within a 12 Mb ROH block. (**E**) Using VirPy, we found 5 β-HPVs, HPV-14, -17, -21, -25, and -47, with β-HPV-14 as the predominant HPV present in the wart. β-HPV-14, -21, and -25 were only present in the wart, whereas β-HPV-47 was also present in the normal-appearing skin samples. Created with BioRender.com. 1000G, 1000 Genomes Project; ExAC, Exome Aggregation Consortium; URR, upstream regulatory region.

We found recurrent *CIB1* mutations in families 11, 12, and 13. The proband of family 11, a 31-year-old man, had an LoF variant of *CIB1*: NM_006384.3, Exon 6, c.548_549dupTT located in a 12 Mb ROH (Figure 2 and Figure 12, A–D). Using VirPy, we found β-HPV-14, -17, -21, -25, and -47, with β-HPV-47 being the predominant HPV subtype present in the wart, BCC, and normal-appearing skin samples of the patient. β-HPV-14, -21, and -25 were present in the wart, whereas only β-HPV-47 and -17 were present in the normal-appearing skin (Figure 12E and Table 3). For the probands of families 12 and 13, we detected β-HPV-17 and β-HPV-5 as the predominant HPV subtypes, respectively (Table 3).

### Predominant forms of HPV infection and new associations of HPV types and subtypes in the cohort of heritable typical EV.

In this study, we analyzed 24 whole transcriptomes of 926 viruses, including 441 HPV subtypes, in the wart and normal-appearing skin of patients with typical EV, using our newly developed, unbiased whole-transcriptomic pipeline, VirPy (11, 16). With this approach for cutaneous virome profiling, we found that β-HPV-14 was the predominant HPV subtype, being detected in 46% of tested samples. This was followed by β-HPV-17, -25, -5, and -22, which were present in 25%, 21%, 21%, and 17% of the tested samples, respectively. At least 1 β-HPV subtype was detected in all of the patients’ biopsy samples.

**Figure 13:**
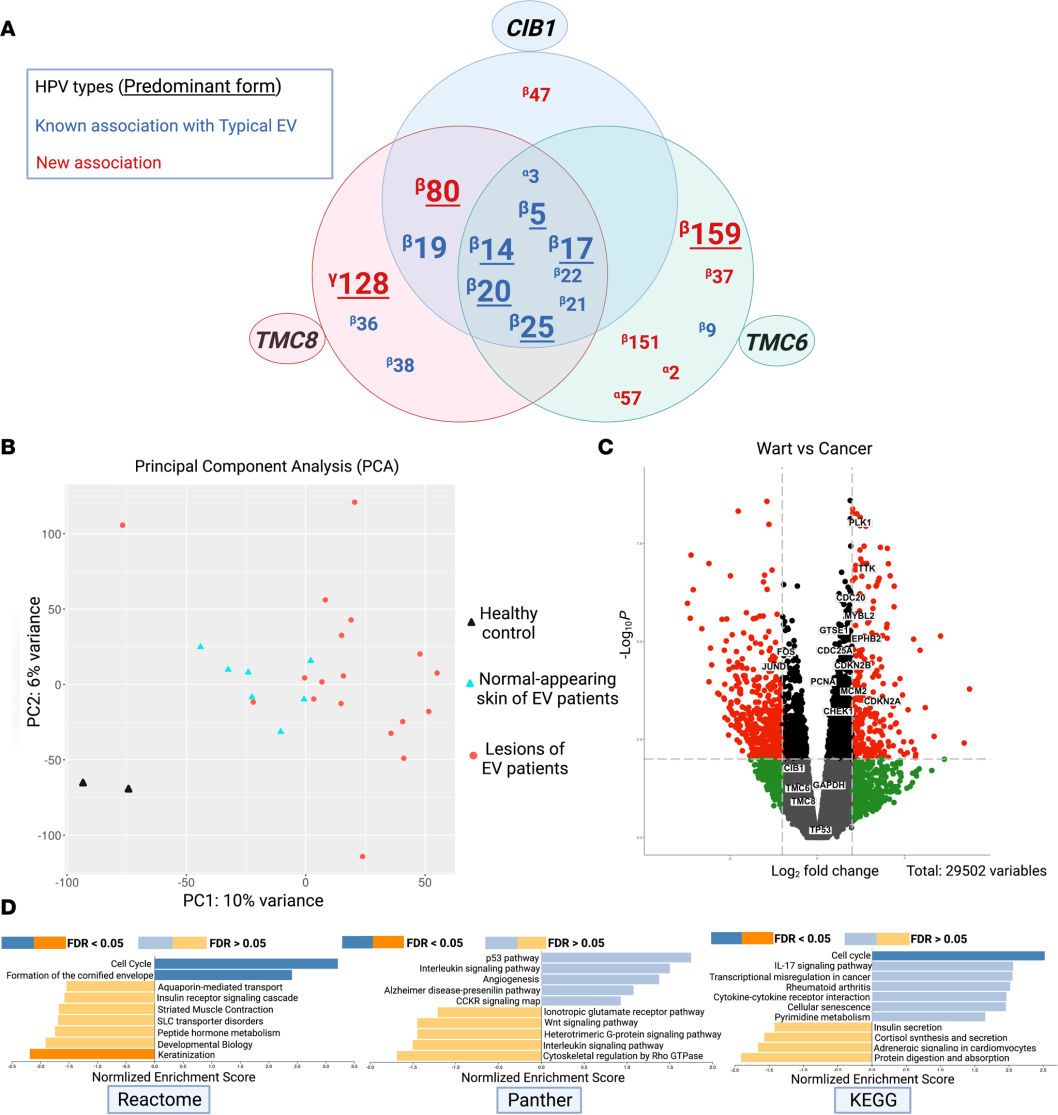
Viral repertoire and transcriptomic analysis by gene set enrichment analysis in patients with EV. (**A**) Twenty different HPVs were detected in patients with typical EV, using unbiased whole-transcriptome sequencing. A Venn diagram was used to visualize the breakdown of the detected viruses in each group of patients with a mutation in 1 of the 3 typical EV genes, *TMC6*, *TMC8*, or *CIB1*. The predominant HPVs are shown with larger font sizes, previously associated HPVs are shown in blue, and potentially novel associations are shown in red. (**B**) A PCA plot of the RNA-Seq data characterizes the trends exhibited by the expression profiles of the skin biopsy specimens of healthy control participants, normal-appearing skin of patients with EV, and warts or cancer lesions of patients with EV. Note the distinct expression profile of the 3 examined groups. (**C**) The volcano plot shows selected DEGs. The log2 fold change indicates the mean expression level for each gene. Each dot represents 1 gene. Black and gray dots represent no significant DEGs between warts and cancer group, and the red dots represent dysregulated genes. (**D**) The GSEA was used for functional assessment to group genes to sets that share common biological functions and was performed using 1808 DEGs in the wart samples versus cancers. Note the dysregulation of cell cycle–related pathways. Created with BioRender.com. 1000G, 1000 Genomes Project; ExAC, Exome Aggregation Consortium; URR, upstream regulatory region.

Additionally, we found new associations of α-HPV-2, α-HPV-57, β-HPV-37, β-HPV-151, and β-HPV-159 with *TMC6* deficiency; γ-HPV-128 and β-HPV-80 with *TMC8* deficiency; and β-HPV-47 and β-HPV-80 with *CIB1* defects (Figure 13A and Tables 1–3). In the tested skin biopsy specimens of patients 1, 12, and 13, only 1 HPV type was detected. In each tested wart biopsy specimen of patients 3, 4, and 17, 2 different HPVs were detected. In the normal-appearing skin biopsy specimens of patients 3 and 4, α-HPV-2, along with β-HPV-9 and -20, were detected. In 1 wart of patient 9, α-HPV-57 and β-HPV-22 and -159, were detected. In the remaining families, at least 3 different HPVs were detected; patients 14 and 16 had 6 and 8 different HPVs, respectively (Tables 1–3 provide the viral counts). Of note, we did not find any definitive correlation between the presence of particular HPV subtypes, the HPV viral load as judged by maximum exon coverage, or co-infections with different HPV subtypes and the degree of phenotypic severity (e.g., the presence or absence of warts, cancer aggressiveness, or histopathological findings).

Given the small sample size of our cohort for each mutation, we could not definitively state a causal link between different HPV subtypes and different clinical presentations. It is also important to note that sampling bias could not be excluded, given that all the control biopsy specimens used for comparison were taken strictly from the forearm or hand of healthy individuals.

### Functional enrichment analysis and annotation of the biological function of TMC6, TMC8, and CIB1 gene mutations and comparison of warts, normal-appearing skin, and healthy control skin.

The potential molecular mechanisms affected by *TMC6*, *TMC8*, and *CIB1* gene defects in the patients’ wart and normal-appearing skin compared with skin samples from 2 healthy control participants were analyzed on the basis of the associated differentially expressed genes (DEGs). Notably, principal component analysis (PCA) demonstrated the consistency of 2 clusters of gene expression profiles in the patients’ lesions (wart and malignant tumors) and normal-appearing skin, compared with the healthy control skin from unrelated individuals in 25 samples (Figure 13B).

Although there were 2 main clusters in the PCA plot of control versus EV samples, a distinction could be made between the normal-appearing skin and lesions (red circles in Figure 13B) overall from the patients, and the normal-appearing skin clusters (blue triangles in Figure 13B) near the unrelated healthy control participants (black triangles in Figure 13B). This suggested that warts have a more distinct biological basis than the normal-looking skin of patients with EV (Figure 13B).

The volcano plot of DEGs between 11 wart and 7 cancer samples of patients with EV (Figure 13C) shows differential expression of cancer-associated genes, such as *FOS*, *JUND*, *PCNA*, *CHEK1,* and *CDKN2A*. The 1,808 dysregulated genes in wart versus cancer samples with *P* < 0.05 and log fold change greater than 2 or less than –2, and common to edgeR and DESeq2, were selected for gene set enrichment analysis (GSEA) by WebGestalt 2019 (http://www.webgestalt.org/option.php). The Reactome database, which provides known biological processes and pathways, showed the 5 cell cycle–related pathways (an average of 5 pathways: normalized enrichment score [NES], 3.2; FDR, <2.2 × 10^–16^; *P* = 0) being positively dysregulated (Figure 13D). The Kyoto Encyclopedia of Genes and Genomes database, which clusters genes on the basis of participation within the same biological process, showed transcriptional dysregulation in cancer (NES, 2.0017; FDR, 0.0651; *P* = 0) and cell cycle (NES, 2.4256; FDR, <2.2 × 10^–16^; *P* = 0.0023) as the most positively enriched pathways in warts compared with normal-appearing skin. Panther, which also captures the collective knowledge represented in biological pathways, showed angiogenesis (NES, 1.41; FDR, 0.253; *P* = 0.039) and p53 signaling (NES, 1.79; FDR, 0.0772; *P* = 0.019) pathways were the most positively enriched (Figure 13D). Collectively, these data confirm the precancerous nature of warts and implicate them in the transformation of normal-appearing skin to the NMSCs observed in all of our patients with *TMC6*, *TMC8*, and *CIB1* mutations.

## Discussion

In this study, we report 5 possibly novel variants in *TMC6*, 1 potentially novel founder mutation in *TMC8*, and 1 founder mutation in *CIB1*. We expanded the total number of identified pathogenic sequence variants in *TMC6*, *TMC8*, and *CIB1* to 14, 11, and 6, respectively (Tables 1–6). We investigated the consequences of disease-associated variant alleles at the mRNA level by RNA-Seq. We report 2 missense variants that were predicted to be disease-causing and to “potentially disrupt the normal splicing events,” (HSF) as judged by several algorithms for evaluating missense changes (*TMC6*: p.Ala297Pro) and the Sashimi plot (*TMC6*: p.Arg551Trp) (Figure 2).

Patients with EV develop HPV-associated malignant lesions at a much higher rate than the general population (7). By using RNA-Seq reads unmapped to human references, we investigated the HPV viral repertoire of 24 skin biopsy specimens from 13 patients. Of the 441 HPV subtypes assessed by VirPy, we found 20 distinct HPVs in our cohort of patients, 8 of which (β-HPV-37, -47, -80, -151, and -159; α-HPV-2 and -57; and γ-HPV-128) were detected for the first time, to our knowledge, in patients with EV. Most patients were solely infected by cutaneous β-HPVs, but 3 were co-infected by low copy numbers of cutaneous or mucosal α-HPVs. γ-HPV was identified as the predominant HPV only once.

In general, the γ- and β-HPVs can be considered commensal organisms of the cutaneous microbiome because their presence can be detected in the majority of healthy individuals. The role of β-HPVs in EV pathogenesis has been well established. There is a report regarding a weak association of γ-HPV-50 with a sporadic case of EV (5). Here, we uncovered that γ-HPV-128 was the predominant subtype in the wart and SCC samples of patient 13 with a *TMC8* mutation.

The protein complex of EVER1-EVER2-CIB1 encoded by the *TMC6*, *TMC8*, and *CIB1* genes in typical EV has a restricted role in infections caused by HPVs lacking E5 expression (15). Therefore, defects in any of these proteins permit the propagation of active HPV infections, which often results in the development of warts.

Patients with EV present with various types of cutaneous malignancies, such as SCC and BCC, that are due to the carcinogenic properties of β-HPVs (6, 15, 17). Nevertheless, the exact pathogenicity of γ-HPVs in patients with EV remains to be explored. In our study, there was a high incidence of early-onset NMSC in more than 80% of our patients with typical EV with different HPVs. Among reported patients with atypical EV found in the literature, 48 of 75 patients had NMSC in their early thirties (mean [SD] age, 31.5 [14.5] years; *n* = 20 women [42%] and *n* = 28 men [58%] (6). One family with a *CIB1* mutation has been reported with basosquamous carcinoma, and pathology revealed infiltrative malignant cells with both basaloid and squamous differentiation (15). It should be noted that cancer formation in patients with EV is age dependent. Also, it is important to highlight that there are reports of patients with EV without any evidence of cancer even in their fifth and sixth decades of life, suggesting that carcinogenesis is not a fixed path in the natural clinical history of patients with EV.

Collectively, we summarized all the previously reported mutations in patients with typical EV, examining the genotype and phenotype correlations, and uncovered the HPV subtypes within skin biopsy specimens. We also reported 7 mutations, which appear not to have been reported previously, in a cohort of 26 patients with typical EV. Finally, knowledge of the mutations and the types of HPVs in EV families, along with the understanding of the associated biological pathways, will guide patients’ treatment and assist in the development of allele-specific therapies for this extremely rare genetic disease.

## Methods

### Recruitment of patients with EV phenotype.

The initial cohort comprised a group of 50 patients: 26 with typical EV and 24 with atypical EV. Diagnostic criteria included the presence of extensive flat or recalcitrant warts and suspicion of viral infections, indicated by the presence of koilocytes in the histopathology of biopsied lesions (Figure 1). The IRB of the Pasteur Institute of Iran approved this study. All patients or the parents or guardians of children gave written informed consent to participate in research and to publish their images and medical histories.

### RNA extraction and whole-transcriptome sequencing.

We isolated RNA from full-thickness (3–5 mm) skin biopsy specimens from the warts and normal-appearing skin of patients. The quality assessment of RNA and whole-transcriptome library preparation were done as previously described (18). For more information, please see Supplementary Materials and Methods, and for technical details, see the report by Youssefian et al. (18).

### Processing of RNA-Seq data: visualization of DEGs.

We analyzed the fastq files of RNA-Seq data and generated PCA plots from the normal-appearing skin of 7 patients and the warts of 18 patients (including 1 biological replicate) with an EV diagnosis and compared them with skin samples derived from 2 unrelated, healthy control participants. Processing of RNA-Seq data was done as previously described (18, 19). Also, GSEA was performed in WebGestalt (20) using 1,087 downregulated and 721 upregulated genes in normal-appearing skin versus warts with at least a 2-fold change. The count data were transformed using EdgeR: log2(counts per million+c), with pseudocount c = 5. For differential expression analysis, limma-voom was used within the iDEP workspace. For more information, please see Supplementary Materials and Methods.

### Variant calling and HM from RNA-Seq data.

RNA-Seq was used as the first-tier method for mutation detection in skin biopsy specimens of probands. We created a pipeline (VirPy) for analyzing RNA-Seq data with both the reference transcriptome and reference genome for variant detection and prioritization, which improves variant calling from RNA-Seq data (18). We performed variant calling and HM as previously described (18). For more information, see Supplementary Materials and Methods, and for technical details, see refs. 21 and 22.

### Viral detection.

VirPy (11) allows for comprehensive analysis of virome profiles in patients with IEI. VirPy uses STAR (23), HISAT2 (24), Samtools (25), eXpress (26), Subread featureCounts (21), FreeBayes (http://arxiv.org/abs/1207.3907), Salmon (22), and SnpEff (27) packages. VirPy begins with read-paired fastq files from RNA-Seq, with quality assessment performed using FastQC. The sequences are trimmed and aligned to the human genome (hg19), using STAR. Unaligned and partially aligned reads are assumed to be nonhuman in origin and represent genetic material from microorganisms. The nonhuman paired mates are extracted and re-aligned using the HISAT2 aligner to a compiled viral genome reference containing 926 viral species obtained from the National Center for Biotechnology Information, including 441 types of HPV (2). Viruses with sufficient quantities of unique reads (i.e., those that are concordantly aligned once without secondary alignments) are outputted from the pipeline. Fifty unique reads were chosen as the default cutoff for visualization; however, this parameter can be modified. The virus-containing file is filtered to contain only concordant pairs. Reads aligned to the viral genome reference are sorted and indexed and can be visualized in Integrative Genomics Viewer; maximum exon coverage for each virus is obtained from the Sashimi plot feature of Integrative Genomics Viewer (28).

### Data availability.

Data sets related to this article can be found at https://submit.ncbi.nlm.nih.gov/subs/sra/ SUB11910228/files in the Sequence Read Archive with submission identification document SUB11910228.

### Statistics.

*P* < 0.05 was considered significant.

### Study approval.

This study was approved by the IRB of the Pasteur Institute of Iran, Tehran, Iran. Written informed consent was obtained from all adult patients and the parents or guardians of children to participate in research and publish their images.

## Author contributions

AHS, LY, VB, EJ, JLC, JU, FV, and HV designed the experiments and prepared the manuscript. AHS, LY, FV, CH, and HV performed the experiments and the statistical analyses. MN, HM, SMB, CH, KN, AH, JSP, FMM, ZS, KKH, MT, NF, SZA, GN, and PM assisted in collecting the samples from patients and healthy control participants. AHS is listed before LY because this study is part of his PhD thesis, and he initiated the work.

## Supplementary Material

Supplemental data

## Figures and Tables

**Table 4:**
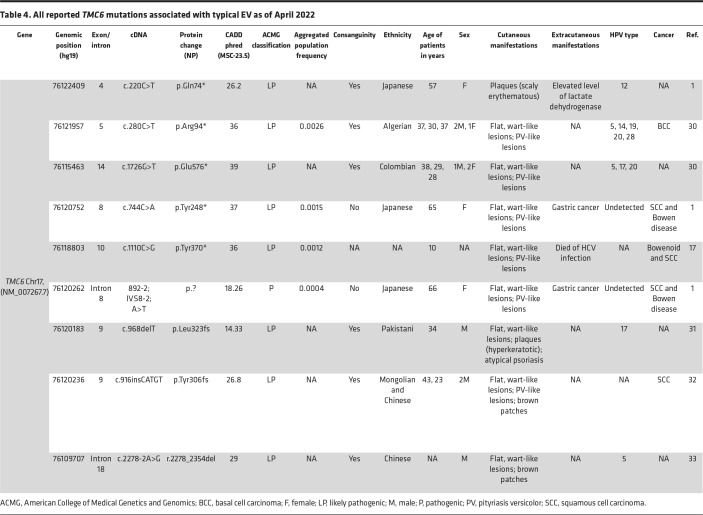
All reported *TMC6* mutations associated with typical EV as of April 2022

**Table 5:**
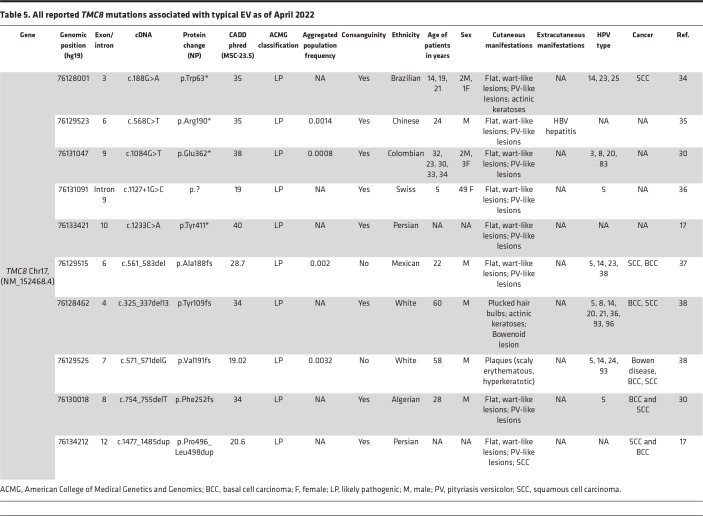
All reported *TMC8* mutations associated with typical EV as of April 2022

**Table 6:**
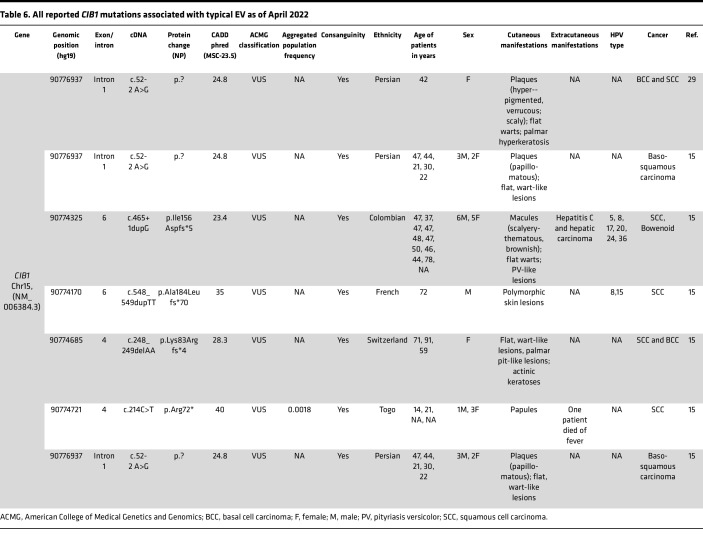
All reported *CIB1* mutations associated with typical EV as of April 2022

**Table 1:**
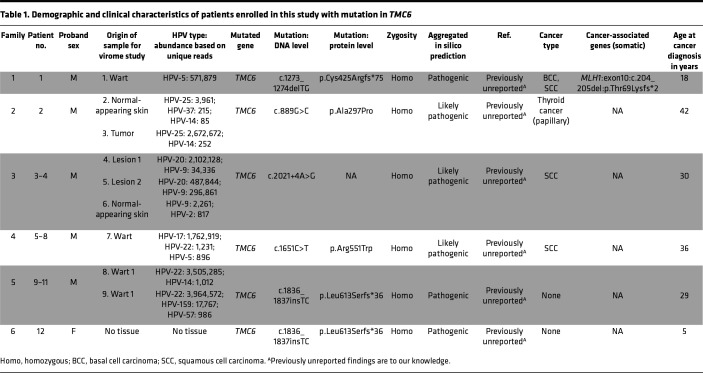
Demographic and clinical characteristics of patients enrolled in this study with mutation in *TMC6*

**Table 2:**
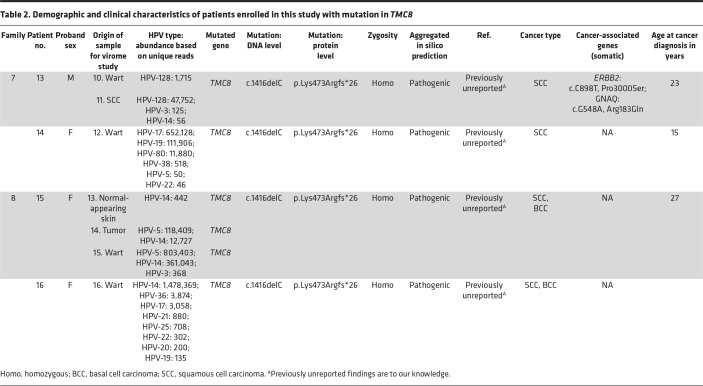
Demographic and clinical characteristics of patients enrolled in this study with mutation in *TMC8*

**Table 3:**
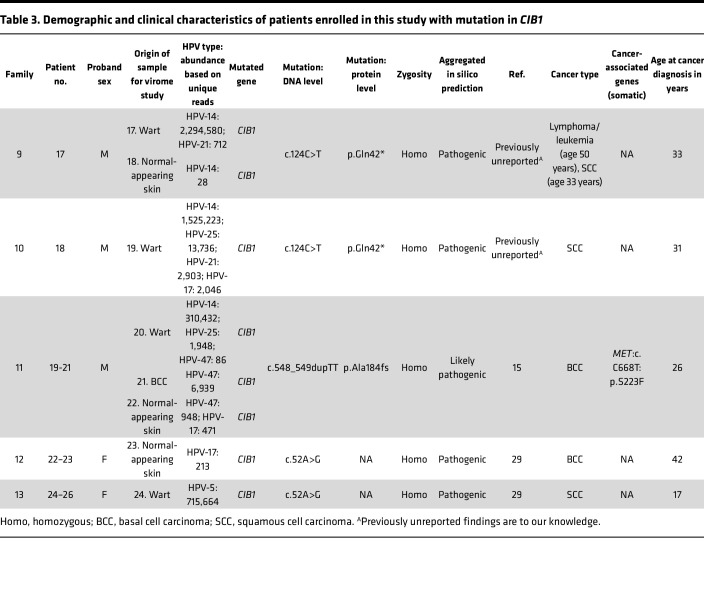
Demographic and clinical characteristics of patients enrolled in this study with mutation in *CIB1*

## Version 1 01/05/2023

In-Press Preview

## Version 2 03/08/2023

Electronic publication
