# Supplementary material for: Whole transcriptome–based skin virome profiling in typical epidermodysplasia verruciformis reveals α-, β-, and γ-HPV infections
Source: JCI Insight. 2023 Mar 8;8(5):e162558. doi: 10.1172/jci.insight.162558 (PMC10077487; doi:10.1172/jci.insight.162558)
Supplement: Supplemental data [file jciinsight-8-162558-s287.pdf]

Supplementary Figure 1

Validation of the VirPy findings by RT-PCR method. We confirmed the presence of positive HPV types in eight EV patient samples using L1- specific primers.

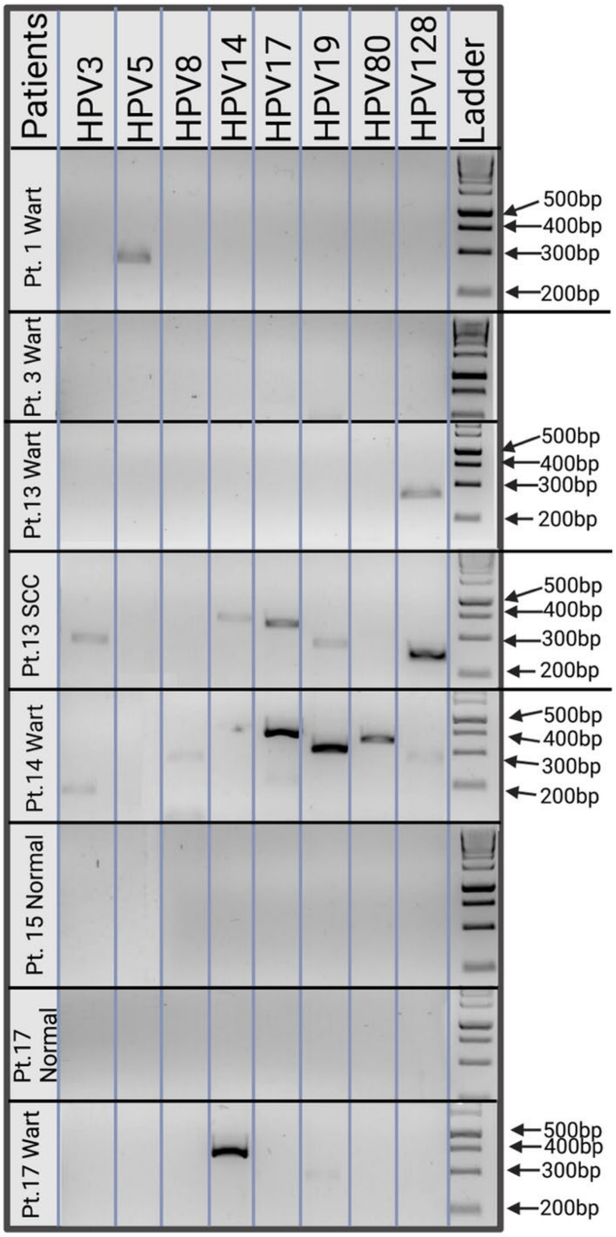

Supplementary Materials and Methods

RNA extraction and whole transcriptome sequencing

RNA was isolated from a full-thickness (3-5 mm) whole-skin biopsy from warts and normal-looking skin using TRIzol® Reagent and quantified on a Nanodrop ND-100 spectrophotometer (Thermo Fisher Scientific, Wilmington, DE), followed by RNA quality assessment on an Agilent 2200 TapeStation (Agilent Technologies, Palo Alto, CA). Following the manufacturer's protocol using TruSeq Standard Total RNA Kit (Illumina, San Diego), 100 ng of total RNA was used for mRNA capture, library preparation, and barcoding at a 4-nmol/L concentration. Using an Illumina NextSeq500 at the Cancer Genomics facility at Thomas Jefferson University, sequencing was performed with 150-bp paired-end chemistry, and a sequencing depth of 50-100 million paired reads per sample was achieved. For technical details see reference.[1]

Processing of RNA-Seq data: Visualization of differentially expressed genes

Read mapping was performed for two controls, seven patients' normal-looking skin (including one biological replicate), and 18 patients' warts with the initial diagnosis of EV, using Hisat2 and the UCSC reference genome hg19. Alignment rates were 91% or greater. Following the mapping phase with STAR[2], the StringTie[3] program was used to assemble the aligned sequences. The outputs of the assembly were later used to extract differentially expressed genes (DEGs). The average number of reads for each sample was ~50 million. StringTie was used to quantify the number of reads mapping to each gene using hg19, and the prepDE.py script provided with StringTie was used to generate a read counts matrix. The matrix of all samples was normalized by the trimmed mean of M values method, and a t-test was performed to identify DEGs between control and patient samples. Count tables were initially analyzed in iDEP4, and the genes were

further analyzed, the data were pre-processed, with a minimum of five counts per million in at least two libraries. The DEGs were selected based on a log2fold-change (FC) >2 or < −3 and a false discovery rate (FDR) <0.05. Significantly meaningful DEGs were enriched by Enrichr5. The DEGs were identified by comparing wart vs cancer samples of EV patients. Heat maps were created using Python. Gene set enrichment analysis (GSEA) was performed in WebGestalt[6] utilizing 1,087 down-regulated and 721 up-regulated genes in wart vs cancer sample with at least two-FC. Principal component analysis (PCA) plots were generated based on three groups consisting of two controls, seven normal-looking skin, and 18 warts/cancer samples (lesions of EV patients). The count data were transformed using EdgeR: log2(CPM+c), with pseudocount c =5. For differential expression analysis, limma-voom was used within the iDEP workspace.

Variant calling and Homozygosity Mapping from RNA-Seq data

RNA-Seq was used as the first-tier method for mutation detection on skin biopsies of probands. We created a novel pipeline for analyzing RNA-Seq data with both the reference transcriptome and reference genome for variant detection and prioritization[7] which improves variant calling from RNA-Seq data.[7] After variant calling, the result files were annotated using Annovar.[8] Prioritization toward identifying rare and pathogenic variants initially focused on exonic sequence variants and removing benign synonymous variants with a Combined Annotation Dependent Depletion (CADD) score of <20. The variants were filtered by including only those with minor allele frequency <0.001 and removing benign variants based on various prediction programs. The loci of putative pathogenic variants were aligned with Runs of Homozygosity (ROHs) determined by Homozygosity Mapping (HM). ROHs were obtained using PLINK from RNA-Seq data by the detailed methods previously described.[1,7]

1. Saeidian AH, Youssefian L, Vahidnezhad H, Uitto J. Research Techniques Made Simple: Whole-transcriptome sequencing by RNA-Seq for diagnosis of monogenic disorders. J Invest Dermatol. 2020;140(6):1117-1126.
2. Dobin A, Davis CA, Schlesinger F, et al. STAR: Ultrafast universal RNA-seq aligner. Bioinformatics. 2013;29(1):15-21.
3. Pertea M, Kim D, Pertea GM, Leek JT, Salzberg SL. Transcript-level expression analysis of RNA-seq experiments with HISAT, StringTie and Ballgown. Nat Protoc. 2016;11(9):1650-1667.
4. Ge SX, Son EW, Yao R. iDEP: an integrated web application for differential expression and pathway analysis of RNA-Seq data. BMC Bioinformatics. 2018;19(1):534.
5. Kuleshov MV, Jones MR, Rouillard AD, et al. Enrichr: a comprehensive gene set enrichment analysis web server 2016 update. Nucleic acids research. 2016;44(W1):W90-97.
6. Wang J, Vasaikar S, Shi Z, Greer M, Zhang B. WebGestalt 2017: a more comprehensive, powerful, flexible and interactive gene set enrichment analysis toolkit. Nucleic acids research. 2017;45(W1):W130-W137.
7. Youssefian L, Saeidian AH, Palizban F, et al. Whole-Transcriptome Analysis by RNA Sequencing for Genetic Diagnosis of Mendelian Skin Disorders in the Context of Consanguinity. Clin Chem. 2021;67(6):876-888.
8. Wang K, Li M, Hakonarson H. ANNOVAR: functional annotation of genetic variants from high-throughput sequencing data. Nucleic acids research. 2010;38(16):e164.
